# Patient-specific musculoskeletal modeling of the hip joint for preoperative planning of total hip arthroplasty: A validation study based on in vivo measurements

**DOI:** 10.1371/journal.pone.0195376

**Published:** 2018-04-12

**Authors:** Maximilian C. M. Fischer, Jörg Eschweiler, Fabian Schick, Malte Asseln, Philipp Damm, Klaus Radermacher

**Affiliations:** 1 Chair of Medical Engineering, Helmholtz-Institute for Biomedical Engineering, RWTH Aachen University, Aachen, Germany; 2 Department for Orthopaedic Surgery, University Hospital RWTH Aachen, Germany; 3 Julius Wolff Institute for Biomechanics and Musculoskeletal Regeneration, Charité - Universitätsmedizin Berlin, Germany; University of Memphis, UNITED STATES

## Abstract

Validation of musculoskeletal models for application in preoperative planning is still a challenging task. Ideally, the simulation results of a patient-specific musculoskeletal model are compared to corresponding in vivo measurements. Currently, the only possibility to measure in vivo joint forces is to implant an instrumented prosthesis in patients undergoing a total joint replacement. In this study, a musculoskeletal model of the AnyBody Modeling System was adapted patient-specifically and validated against the in vivo hip joint force measurements of ten subjects performing one-leg stance and level walking. The impact of four model parameters was evaluated; hip joint width, muscle strength, muscle recruitment, and type of muscle model. The smallest difference between simulated and in vivo hip joint force was achieved by using the hip joint width measured in computed tomography images, a muscle strength of 90 N/cm^2^, a third order polynomial muscle recruitment, and a simple muscle model. This parameter combination reached mean deviations between simulation and in vivo measurement during the peak force phase of 12% ± 14% in magnitude and 11° ± 5° in orientation for one-leg stance and 8% ± 6% in magnitude and 10° ± 5° in orientation for level walking.

## 1 Introduction

Total hip arthroplasty (THA) is the most frequently performed joint replacement surgery [1] and primary THA is one of the most successful interventions in orthopedic surgery measured in terms of cost-effectiveness and quality of life outcome [2]. Although the revision rate of THA stagnates [3], the economic burden of revision THA is notable due to the high and growing number of primary THA. Revision THA is more expensive and has a lower quality of life outcome than primary THA [4,5]. In 2001, Maloney estimated a cost-savings effect above $30 million per year in the US when a 5% reduction of the revision rate is considered [6].

Misalignment of prosthetic components was identified as a major risk factor for aseptic loosening, the main reason for revision THA, also associated with implant wear [7]. To minimize wear, high stresses have to be avoided. Therefore, the implant’s alignment should be planned preoperatively with regard to minimal loading of the components and optimal force orientation for demanding activities of daily living. In particular, excessive edge loading of the acetabular inlay has to be prevented and the edge loading risk is considered one of the major criteria to define a patient-specific load-based cup alignment [8,9]. [10]

In order to estimate the loading of the implant preoperatively, the magnitude and orientation of the hip joint force (HJF) have to be approximated. The HJF can be utilized within a preoperative planning process to define the edge loading risk [11,12]. With this information, a load-based target zone can be calculated [11]. The highest loads in the hip joint occur in the upright position during level walking, one-leg stance, stair climbing, and jogging [10]. Thus, it is reasonable to weight peak forces higher when calculating the load-based target zone in order to avoid permanent edge loading caused by large forces during activities of daily living Of course, other criteria, like range of motion, should also be considered for the calculation of a patient-specific target zone [9]. Consequently, the surgeon can be informed preoperatively about the maximum acceptable surgical error, or in the case of navigated surgery, he could be intraoperatively informed about the current position within the patient-specific target zone.

However, magnitude and orientation of the HJF are inter-individually different and change during activities of daily living [10]. The calculation of the HJF, therefore, has to be adapted to the individual characteristics of the patient to estimate edge loading.

Musculoskeletal models (MSM) are a common method to study the mechanics of joints [13], and a patient-specific adapted MSM offers the possibility to approximate the individual HJF for activities of daily living [14]. Presently, the implementation of MSM into the preoperative planning process is still difficult, because, among other reasons, the validation of MSM is a challenging task [15,16]. Pierrepont et al. reported on the use of a MSM within the framework of patient-specific THA planning. However, no information was given concerning the validation of their proprietary MSM [12].

In order to validate the use of MSM, the results of a subject-specific simulation should be compared to the experimental in vivo data of the same subject. Instrumented prostheses have been used to measure in vivo joint forces in patients who have undergone an artificial joint replacement. The force and torque data is transmitted via telemetry during different activities, which are recorded by a motion tracking system [17]. To the knowledge of the authors, only four MSM have been validated with motion tracking data and the corresponding in vivo data of the hip joint [18–21]. All studies used the data from a maximum of four subjects from the HIP98-database [22]. In three of these studies, anthropometric data derived from computed tomography (CT) scans, X-ray images, and motion tracking data was used to patient-specifically scale the MSM [18,19,21].

The aim of this study was to validate an adaptable MSM of the lower body for preoperative planning in THA. The simulation results of the patient-specific adapted MSM were compared to the corresponding in vivo measurements from the OrthoLoad database (www.orthoload.com) for two activities: one-leg stance and level walking. With 10.2%, level walking is the third most frequent activity of THA patients following sitting (44.3%) and standing (24.5%) [23]. However, as a single-legged exercise, the peak forces of level walking are 1.5 to 2.5 times higher than the peak forces of double-legged exercises, like sitting and standing [10]. Additionally, one-leg stance was analysed because it is used as a static surrogate for the peak force phase (PFP) during level walking in both analytical models for HJF estimation [24] and for preoperative planning of THA [25].

The impact of four modeling parameters on the simulated HJF was evaluated; the hip joint width (HJW), the muscle strength, the muscle recruitment, and the muscle model.

The HJW affects the lever arms of the muscles spanning over the hip joint. It has been demonstrated that medialization of the hip joint center decreases the HJF [26,27]. If the patient-specific scaling of the model, purely based on the motion tracking data, overestimates the HJW, then the simulated HJF may exceed the in vivo HJF.

A wide range of muscle strengths are reported depending on the type of skeletal muscles and the loading conditions [28]. Due to the fact that it may be difficult and inefficient to assess the patient-specific data for muscle strength in a clinical workflow, an optimal value has to be found that coincides with all patients.

While a general muscle recruitment criterion based on the understanding of the central nervous system will not be found in the near future, multiple recruitment criteria based on energy conservation of the muscles have been proposed [29]. As the choice of the recruitment criteria may be activity dependent [30], two commonly used criteria, polynomial and strict min/max [31], are compared in this study evaluating the effect on the simulated HJF.

Since the muscle model is the interface between the muscle recruitment criteria and the articulating segments, it should be selected depending on the objective of the MSM [32]. This study investigates if a complex Hill-type muscle model is necessary to improve simulation results.

## 2 Material & methods

### 2.1 Patient-specific data

Data from ten patients of the OrthoLoad database (two females and eight males) with a mean age of 56.9 ± 5.5 years, mean body weight of 90 ± 13 kg and mean body height of 1.74 ± 0.06 m was used in this study (Table 1). All subjects suffered from osteoarthritis and underwent THA. The implanted, instrumented Hip III prosthesis monitors forces and torques acting on the head of the femoral stem [17]. Motion capture data (VICON Metrics, Oxford, UK) and force plate data (AMTI, Watertown, USA) of one-leg stance and level walking was recorded in order to take into account the patient-specific motion behavior during simulation. Simultaneously, the in vivo HJF was measured by the instrumented prosthesis and transmitted via telemetry (S1 Table). The HJW was defined as the distance between the hip joint centers. The hip joint centers were manually identified with the software ITK-SNAP 2.4.0 (www.itksnap.org) on postoperative CT scans of each subject.

**Table 1:** Anthropometric data of the ten patients with the instrumented Hip III implant of the OrthoLoad database.

Patient ID	Side	Age	Sex	Body weight [kg]	Body height [m]	BMI [kg/m^2^]	HJW [mm]
H1L	L	55	m	77.5	1.78	24.5	166
H2R	R	61	m	78.2	1.72	26.4	160
H3L	L	59	m	90.9	1.68	32.2	174
H4L	L	50	m	81.1	1.78	25.6	174
H5L	L	62	f	87.2	1.68	30.9	186
H6R	R	68	m	83.1	1.76	26.8	157
H7R	R	52	m	93.4	1.79	29.1	172
H8L	L	55	m	85.2	1.78	26.9	180
H9L	L	54	m	122.0	1.81	37.2	174
H10R	R	53	f	101.4	1.62	38.6	176

### 2.2 Musculoskeletal simulation

The lower body MoCap-Model from the AnyBody Managed Model Repository of the AnyBody Modeling System (AnyBody Technology, Aalborg, Denmark) was used [33,34]. AnyBody is a multi-body simulation software that offers the choice between different muscle recruitment criteria to solve the well-known muscle redundancy problem. It is also possible to use different muscle models to simulate the behavior of the muscles under different loading conditions. The lower body MoCap-Model consists of the trunk and the legs. Head, thorax and lumbar spine are part of the trunk. The head is connected to the thorax by a revolute joint with one degree of freedom. The thorax is connected to a detailed lumbar spine model with seven spherical joints, each with three degrees of freedom. The lumbar spine model contains eleven muscles subdivided into 203 fascicles and a model of the abdominal pressure [35]. The legs are based on cadaver data from the Twente Lower Extremity Model [36]. The segments of each leg are connected by five joints: the hip joint and both parts of the knee and ankle joints. The hip joint is a spherical hip joint with three degrees of freedom. The knee consists of two revolute joints for tibiofemoral and patellofemoral flexion and extension. The ankle is composed of two revolute joints for flexion and extension as well as eversion and inversion. Each leg contains 56 muscles with 159 fascicles [37].

**Fig 1:**
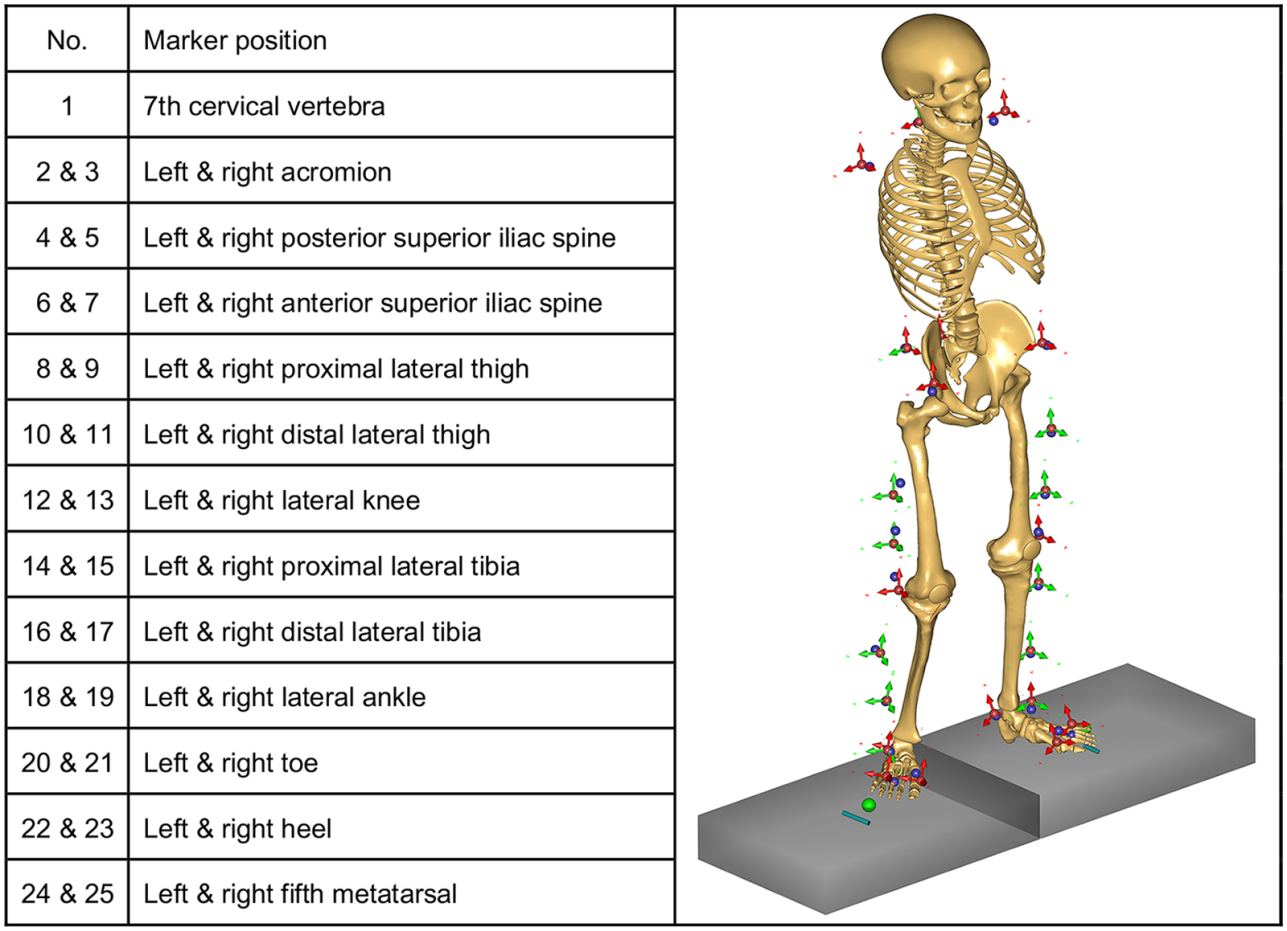
Marker locations of the motion capture data used for the patient-specific parameter optimization of the model.

The simulation with the MoCap-Model consists of two steps. In the first step, the model is patient-specifically adapted using a method called linear scaling described in detail by Lund et al. [38]. The segment lengths of the model are scaled during an optimization procedure based on the skin marker trajectories of the motion capture trial [39]. The perpendicular directions are scaled by a scaling law taking into account body height and body weight [40]. As proposed by AnyBody, a weighting function based on the residuals of the markers was implemented to handle marker dropouts during the motion capture. Twenty-five markers were used as input for the optimization (Fig 1). The parameter optimization is only related to the kinematics and determines the individual segment length ratios and joint angles.

The patient-specific scaled model, the individual joint angles, and force plate data serve as input for the second step of simulation. During the inverse dynamics simulation, the muscle activations, muscle forces, and the joint reaction forces are computed. The simulations were performed on a common desktop computer (Intel Core i7 4770 @ 3.40 Ghz, 16 GB RAM).

The impact of four simulation parameters on the HJF was investigated: the HJW, the muscle strength, the muscle recruitment, and the muscle model.

1. For the HJW, two cases were simulated: a Coordinate 3D-based HJW (C3D-HJW) optimized solely by the skin marker trajectories in the first step of the simulation, and a fixed CT-based HJW (CT-HJW) measured in the postoperative CT scans. The fixed CT-HJW is not altered during the parameter optimization based on the skin markers.
2. Reported strength of skeletal muscles ranges from 20 N/cm^2^ to 100 N/cm^2^ depending on the cross-sectional area of the muscle [28]. Two values for the muscle strength were compared in the simulations: 40 N/cm^2^, similar to the value used in comparable studies [20,21], and 90 N/cm^2^, the default value used by AnyBody.
3. A well-known drawback of complex MSM using the inverse dynamics approach is the underdetermined set of equations of the musculoskeletal system. The term “muscle redundancy problem” describes the fact that the number of muscles exceeds the degrees of freedom of the MSM. The muscle redundancy problem is solved through numerical optimization. The objective function of the optimization procedure is called “muscle recruitment criterion” [30]. In this study, a polynomial, third power muscle recruitment criterion (PN) was compared with a strict min/max formulation (MM), which is equivalent to an infinite power of the polynomial criterion [31]. With increasing power, the load is shared more equally between the muscles, while muscle fatigue is minimized [29].
4. The difference between the use of a simple muscle model and a Hill-type muscle model for the legs was investigated. The simple muscle model has a constant strength and no passive elements. It is independent of muscle length and contraction velocity; therefore it does not consider any contraction dynamics [41,42]. In contrast, the Hill-type muscle model considers a contractile element, a non-linear serial elastic element, and a non-linear parallel elastic element [32]. The parameters of the Hill-type muscle model are taken from the Twente Lower Extremity Model and other sources [36,43,44]. For the trunk, only the simple muscle model is used [35].

### 2.3 Model validation

To compare the simulated HJF with the in vivo HJF, both forces have to be presented in the same coordinate system. By default, AnyBody calculates the force in the femoral coordinate system recommended by the International Society of Biomechanics [45], while the OrthoLoad database uses a similar coordinate system but with a slightly different orientation. Therefore, the simulated HJF was transformed into the femoral coordinate system used by the OrthoLoad database [10]. All forces were normalized by the subject’s body weight for a standardized comparison.

**Fig 2:**
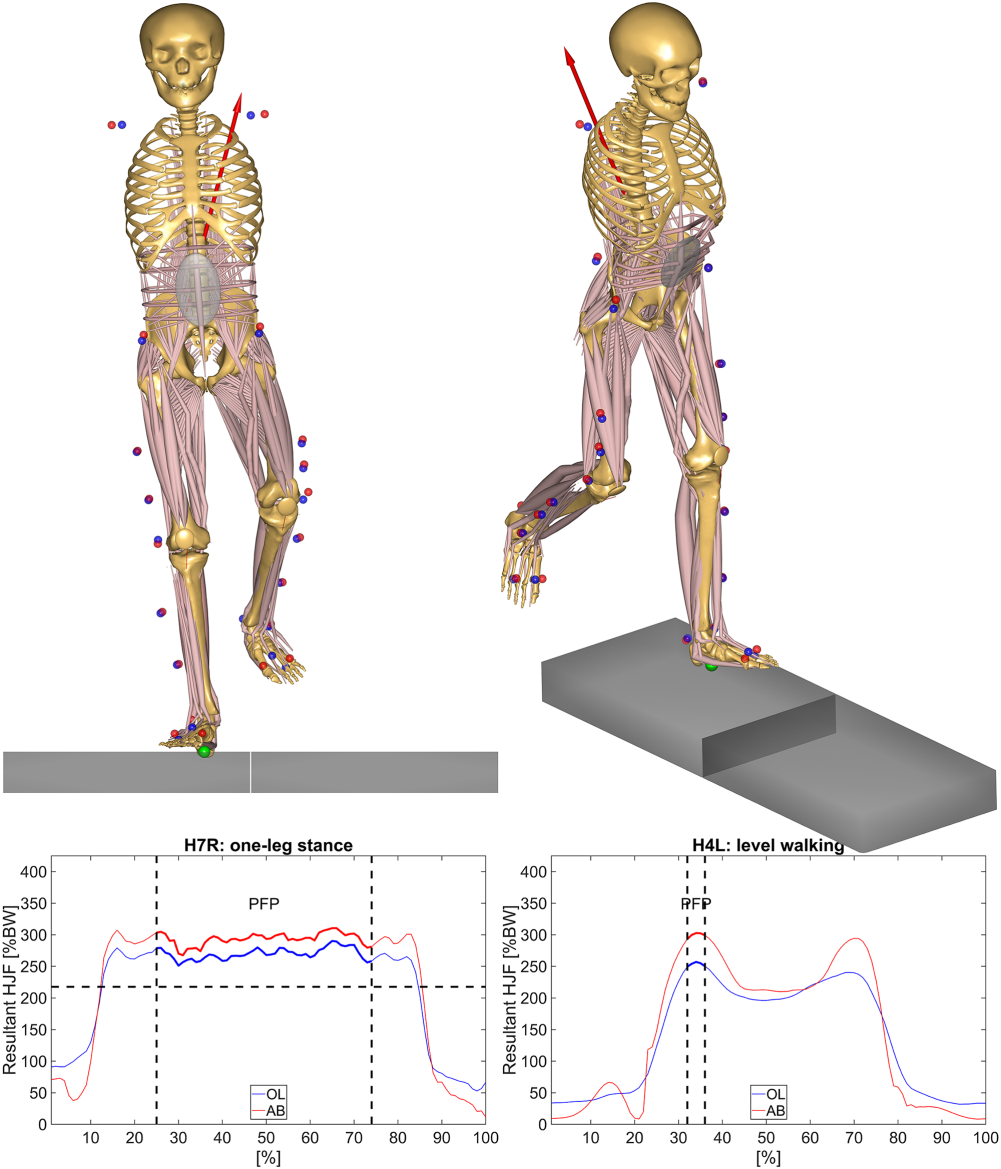
The comparison of the simulated (AnyBody, AB) and in vivo (OrthoLoad, OL) resultant HJF for one-leg stance (left) and level walking (right). The PFP is illustrated by the two dashed, vertical lines. For one-leg stance (left), the 75% threshold of the maximum resultant HJF to identify the plateau phase is illustrated by the dashed, horizontal line.

Four error metrics were used to quantify the deviation between simulated and in vivo HJF of each parameter combination:

- Mean absolute percentage error of the peak force phase (MAPE^PFP^) in percent (%): for one-leg stance, the plateau phase of the resultant HJF was identified within the in vivo data by selecting all values above 75% of the maximum resultant HJF (Fig 2, left). The PFP was defined as the middle of the plateau phase by omitting 25% of beginning and the end of the motion cycle for lifting and lowering of the leg. For level walking, the force peak during contralateral toe off was identified and the PFP was defined as 2.5% of the gait cycle before and after the force peak (Fig 2, right). Then, the percentage deviation between simulated and in vivo PFP was calculated (PD^PFP^) and the mean of the absolute values of all subjects was taken.
- Mean angular deviation of the peak force phase (MAD^PFP^) in degree (°): the angular deviations between the direction of the simulated and the in vivo HJF vector of the PFP were calculated for flexion-extension (MAD^PFP^_FE_), for adduction-abduction (MAD^PFP^_AA_), internal-external rotation (MAD^PFP^_IE_), and in 3D (MAD^PFP^_3D_). Then, the mean of the absolute values of all subjects was taken.
- Root mean square error (RMSE) in percentage of body weight (%BW): the RMSE was calculated for the three components of the HJF in medial-lateral (RMSE_ML_), posterior-anterior (RMSE_PA_) and inferior-superior (RMSE_IS_) direction as well as for the resultant HJF (RMSE_R_) from beginning to end of the motion cycle.
- Squared Pearson correlation coefficient (r^2^): r^2^ was calculated between simulated and in vivo HJF for the three components of the HJF (r^2^_ML_, r^2^_PA_, r^2^_IS_) and the resultant HJF (r^2^_R_) from beginning to end of the motion cycle. Significance of the correlation was tested with a significance level of 0.05.

Subsequently, the parameter combination with the smallest MAPE^PFP^, the smallest sum of RMSE_ML_, RMSE_PA_ and RMSE_IS_, as well as the smallest RMSE_R_ was selected for a further in-depth analysis. Mean and standard deviation (SD), as well as median and interquartiles of each component of the simulated HJF, the in vivo HJF, and the mean absolute error (MAE) of the simulated HJF, was calculated for the full motion cycle. Linear correlation analysis was performed to investigate intercept and slope. The differences between simulated and in vivo HJF were tested for normal distribution using the Kolmogorov-Smirnov test. As the test for normal distribution was rejected at a significance level of 0.05 for all components and the resultant of the HJF, non-parametric statistics were used in the further analysis. Bland-Altman plots were generated including the limits of agreement (LOA), defined as 1.45 times the interquartile range (IQR), and the significance of the difference between simulated and in vivo HJF was tested using the Mann-Whitney U-test with a significance level of 0.05. Bland-Altman plots are an established method to compare two measurements techniques, in this case the AnyBody simulation and the OrthoLoad in vivo measurements. The difference between the simulated and the in vivo HJF is plotted against the reference method, the in vivo HJF [46].

## 3 Results

The variation of the four investigated parameters adds up to 16 different parameter combinations for 10 subjects and two activities resulting in 320 simulations. For all simulations, the mean absolute residuals of the parameter optimization, the first simulation step, are 1%BW ± 2%BW in medial-lateral direction, 3%BW ± 4%BW in posterior-anterior direction and 14%BW ± 11%BW in inferior-superior direction. The results of the four error metrics for the inverse dynamics simulations are presented in Table 2. Incomplete simulations were excluded from the analysis. Of the 320 simulations, 17 were unsuccessful. Each incomplete simulation used the MM recruitment in combination with the simple muscle model.

**Table 2:** Deviations between simulated and in vivo HJF for one-leg stance and level walking for each combination of the four investigated parameters. Only successful simulations were included. The parameter combinations with the smallest MAPE^PFP^, the smallest sum of RMSE_ML_, RMSE_PA_ and RMSE_IS_, as well as the smallest RMSE_R_ are marked in grey.

	Hip Joint Width	Muscle Strength [N/cm^2^]	Muscle Recruitment	Muscle Model	Successful Simulations	MAPE^PFP^ [%]	MAD^PFP^ [°]				RMSE [%BW]				r^2^ (p<0.05)			
					n		FE	AA	IE	3D	ML	PA	IS	R	ML	PA	IS	R
**One-leg stance**	**C3D**	**40**	**PN**	**Simple**	10	13 ± 15	5 ± 3	10 ± 5	11 ± 7	11 ± 4	55	23	32	44	0.48	0.02	0.86	0.84
				**Hill**	10	27 ± 12	4 ± 3	10 ± 4	11 ± 10	11 ± 4	69	21	49	70	0.45	0.04	0.9	0.89
			**MM**	**Simple**	9	69 ± 84	8 ± 5	10 ± 5	15 ± 9	12 ± 5	139	74	161	213	0.24	0.1	0.35	0.3
				**Hill**	10	61 ± 53	6 ± 5	7 ± 4	14 ± 11	10 ± 4	90	57	137	165	0.36	0.11	0.59	0.54
		**90**	**PN**	**Simple**	10	13 ± 14	5 ± 3	10 ± 5	11 ± 8	11 ± 4	55	23	31	43	0.48	0.03	0.87	0.85
				**Hill**	10	31 ± 12	4 ± 3	10 ± 4	10 ± 10	11 ± 4	73	22	57	79	0.45	0.05	0.9	0.89
			**MM**	**Simple**	5	103 ± 83	10 ± 3	12 ± 4	15 ± 6	14 ± 4	172	79	190	256	0.33	.01*	0.42	0.37
				**Hill**	10	61 ± 43	5 ± 4	8 ± 4	14 ± 10	10 ± 4	92	48	131	158	0.39	0.1	0.66	0.63
	**CT**	**40**	**PN**	**Simple**	10	12 ± 14	5 ± 3	10 ± 5	11 ± 8	11 ± 5	53	23	31	42	0.55	0.01	0.87	0.85
				**Hill**	10	26 ± 10	4 ± 3	10 ± 4	11 ± 10	11 ± 4	67	22	44	65	0.52	0.03	0.92	0.92
			**MM**	**Simple**	10	63 ± 81	7 ± 5	9 ± 5	14 ± 9	11 ± 5	128	68	154	201	0.27	0.14	0.35	0.3
				**Hill**	10	51 ± 32	5 ± 3	7 ± 4	13 ± 9	9 ± 3	81	38	109	132	0.48	0.1	0.75	0.72
		**90**	**PN**	**Simple**	10	12 ± 14	5 ± 3	10 ± 5	11 ± 8	11 ± 5	53	23	30	41	0.55	0.02	0.87	0.86
				**Hill**	10	30 ± 10	4 ± 3	10 ± 4	11 ± 10	11 ± 4	71	22	52	74	0.53	0.04	0.92	0.91
			**MM**	**Simple**	6	85 ± 82	8 ± 4	12 ± 4	17 ± 8	15 ± 3	155	70	173	230	0.39	0.06	0.37	0.33
				**Hill**	10	56 ± 33	5 ± 3	8 ± 4	13 ± 9	9 ± 3	87	38	116	141	0.47	0.08	0.75	0.72
**Level walking**	**C3D**	**40**	**PN**	**Simple**	10	10 ± 8	5 ± 3	9 ± 4	8 ± 6	10 ± 4	45	18	34	45	0.47	0.51	0.88	0.88
				**Hill**	10	24 ± 10	4 ± 3	11 ± 6	11 ± 9	12 ± 5	48	24	46	59	0.52	0.38	0.87	0.87
			**MM**	**Simple**	10	30 ± 21	5 ± 4	9 ± 5	10 ± 7	11 ± 5	80	46	77	107	0.31	0.04	0.79	0.73
				**Hill**	10	39 ± 13	5 ± 3	13 ± 7	13 ± 9	14 ± 6	77	58	99	127	0.29	0.03	0.7	0.65
		**90**	**PN**	**Simple**	10	10 ± 8	5 ± 3	9 ± 4	8 ± 6	10 ± 4	45	17	33	43	0.47	0.54	0.89	0.88
				**Hill**	10	29 ± 10	4 ± 3	11 ± 6	11 ± 9	12 ± 5	49	18	46	58	0.52	0.53	0.88	0.88
			**MM**	**Simple**	9	25 ± 15	4 ± 3	9 ± 5	9 ± 7	10 ± 5	70	33	61	84	0.33	0.18	0.85	0.81
				**Hill**	10	44 ± 15	6 ± 4	13 ± 7	13 ± 9	14 ± 6	75	44	89	113	0.32	0.08	0.75	0.72
	**CT**	**40**	**PN**	**Simple**	10	8 ± 6	5 ± 3	9 ± 4	8 ± 6	10 ± 5	44	17	31	41	0.46	0.56	0.9	0.89
				**Hill**	10	24 ± 10	4 ± 3	11 ± 6	11 ± 9	11 ± 6	46	22	43	54	0.53	0.42	0.89	0.89
			**MM**	**Simple**	9	31 ± 18	6 ± 4	10 ± 4	11 ± 7	12 ± 4	83	46	80	110	0.33	0.05	0.77	0.72
				**Hill**	10	36 ± 9	5 ± 3	12 ± 7	12 ± 8	13 ± 6	72	54	92	117	0.34	0.04	0.72	0.68
		**90**	**PN**	**Simple**	10	8 ± 6	5 ± 3	9 ± 4	8 ± 6	10 ± 5	44	16	31	40	0.46	0.58	0.9	0.89
				**Hill**	10	28 ± 10	4 ± 3	11 ± 6	11 ± 9	11 ± 5	47	17	43	54	0.53	0.58	0.89	0.9
			**MM**	**Simple**	7	30 ± 19	6 ± 3	10 ± 5	8 ± 5	11 ± 5	79	37	71	100	0.44	0.11	0.8	0.75
				**Hill**	10	41 ± 10	5 ± 3	12 ± 7	13 ± 8	13 ± 6	72	42	84	108	0.35	0.09	0.76	0.73

For the resultant HJF, MAPE^PFP^_R_ ranges from 8% to 103%, mean MAD^PFP^_3D_ from 9° to 15°, RMSE_R_ from 34%BW to 167%BW, and r^2^_R_ from 0.3 to 0.92 for all parameter combinations. The combinations of the PN recruitment and the simple muscle model result in the smallest MAPE^PFP^ and RMSE_R_ without taking into account activity, muscle strength or HJW. For the PN recruitment and the simple muscle model, MAPE^PFP^ ranges from 8% to 13%, MAD^PFP^_3D_ from 10° to 11°, RMSE_R_ from 40%BW to 45%BW, and r^2^_R_ from 0.84 to 0.89. For the simulations with PN recruitment and simple muscle model, the lowest RMSE appears in posterior-anterior direction (16%BW to 21%BW) and the highest RMSE in medial-lateral direction (44%BW to 53%BW) for both activities. The high RMSE in medial-lateral direction corresponds to the higher angular deviations for adduction-abduction and internal-external rotation than for flexion-extension. MAD^PFP^_FE_ is 5° while MAD^PFP^_AA_ ranges from 9° to 10° and MAD^PFP^_IE_ from 8° to 11°.

For all simulations, correlations between simulated and in vivo HJF were significant (p<0.05). r^2^_ML_ ranges from 0.24 to 0.55, r^2^_PA_ ranges from 0.03 to 0.58 and r^2^_IS_ ranges from 0.3 to 0.92. Overall r^2^ values in medial-lateral and inferior-superior direction are higher for PN recruitment than r^2^ values for MM recruitment. For PN recruitment r^2^_ML_ ranges from 0.45 to 0.58 and r^2^_IS_ ranges from 0.86 to 0.92, while for MM recruitment r^2^_ML_ ranges from 0.24 to 0.48 and r^2^_IS_ ranges from 0.35 to 0.85. This corresponds to the results of the RMSE. For PN recruitment RSME_ML_ ranges from 44%BW to 73%BW and RMSE_IS_ ranges from 30%BW to 57%BW, while for MM recruitment RMSE_ML_ ranges from 70%BW to 172%BW and RMSE_IS_ ranges from 61%BW to 190%BW.

For the two activities, the parameter combination with the smallest MAPE^PFP^, the smallest sum of RMSE_ML_, RMSE_PA_ and RMSE_IS_, as well as the smallest RMSE_R_ were selected from Table 2 to be examined the in detail. In Table 3, the results, averaged over the full motion cycle, for CT-HJW, 90 N/cm^2^ muscle strength, PN recruitment, and simple muscle model are presented for each subject separately. For the in vivo measurements, the largest component of the HJF is the inferior-superior direction followed by the medial-lateral component and the posterior-anterior component. For both activities, the highest mean absolute error (MAE) between simulated and in vivo HJF occurs in the medial-lateral direction, followed by the inferior-superior and the posterior-anterior direction. For one-leg stance, the subjects with a MAE above 50%BW are H3L, H4L, H5L, H9L and H10R in the medial-lateral direction and H10R in the inferior-superior direction. For level walking, subject H3L has a MAE above 50%BW in the medial-lateral direction. Subject H10R shows high overestimation of the resultant HJF with a MAE above 50% for one-leg stance as well as for level walking. MAE of all subjects for the resultant HJF is 29%BW. The individual motion cycles of each subject can be found in the supporting information (S1 Fig).

**Table 3:** Individual results for one-leg stance and level walking averaged over the full motion cycle for CT-HJW, 90 N/cm^2^ muscle strength, PN recruitment, and simple muscle model. Mean and standard deviation (SD) are presented in %BW for the AnyBody (AB) simulations, the OrthoLoad (OL) in vivo measurements and the MAE of the AnyBody simulations in each direction of the HJF. MAE above 50%BW are marked in grey.

		[%BW]	H1L	H2R	H3L	H4L	H5L	H6R	H7R	H8L	H9L	H10R	Mean ± SD
**One-leg stance**	**ML**	**AB**	45 ± 20	94 ± 36	95 ± 32	138 ± 45	125 ± 74	74 ± 22	99 ± 47	88 ± 41	103 ± 54	133 ± 54	99 ± 52
		**OL**	59 ± 10	61 ± 20	28 ± 6	73 ± 19	88 ± 31	60 ± 15	71 ± 19	68 ± 24	51 ± 22	62 ± 17	62 ± 24
		**MAE**	14 ± 11	34 ± 15	67 ± 26	65 ± 24	55 ± 22	17 ± 6	38 ± 12	24 ± 12	54 ± 30	73 ± 36	44 ± 30
	**PA**	**AB**	-17 ± 5	-29 ± 13	-31 ± 11	-25 ± 7	-42 ± 21	-18 ± 6	-37 ± 20	-16 ± 7	-35 ± 21	-15 ± 5	-27 ± 16
		**OL**	-23 ± 5	-15 ± 7	-26 ± 9	-15 ± 6	-4 ± 4	-31 ± 9	-5 ± 3	-2 ± 4	-9 ± 7	-1 ± 2	-13 ± 12
		**MAE**	7 ± 3	14 ± 8	5 ± 3	10 ± 4	38 ± 18	12 ± 4	34 ± 17	15 ± 8	26 ± 14	13 ± 7	17 ± 15
	**IS**	**AB**	-152 ± 51	-200 ± 74	-196 ± 63	-241 ± 72	-227 ± 112	-181 ± 60	-206 ± 90	-174 ± 78	-191 ± 97	-223 ± 87	-199 ± 84
		**OL**	-172 ± 50	-212 ± 76	-213 ± 64	-211 ± 59	-249 ± 98	-187 ± 66	-208 ± 78	-187 ± 78	-215 ± 99	-177 ± 54	-203 ± 77
		**MAE**	20 ± 12	17 ± 11	17 ± 7	33 ± 14	22 ± 36	12 ± 10	10 ± 12	14 ± 9	24 ± 12	54 ± 23	22 ± 21
	**R**	**AB**	159 ± 55	223 ± 83	220 ± 71	279 ± 85	264 ± 135	197 ± 64	232 ± 103	195 ± 88	220 ± 112	260 ± 102	225 ± 99
		**OL**	184 ± 50	221 ± 79	217 ± 65	224 ± 62	264 ± 102	199 ± 67	221 ± 80	199 ± 81	221 ± 101	188 ± 56	214 ± 79
		**MAE**	24 ± 13	14 ± 11	9 ± 7	58 ± 22	42 ± 28	10 ± 10	26 ± 10	11 ± 9	17 ± 9	79 ± 37	29 ± 29
**Level walking**	**ML**	**AB**	45 ± 25	60 ± 39	77 ± 58	68 ± 50	87 ± 65	72 ± 53	70 ± 43	59 ± 43	59 ± 42	80 ± 58	68 ± 50
		**OL**	53 ± 15	36 ± 17	27 ± 10	44 ± 19	58 ± 28	54 ± 20	50 ± 16	57 ± 29	40 ± 17	59 ± 12	48 ± 22
		**MAE**	16 ± 10	27 ± 21	54 ± 51	33 ± 23	43 ± 29	33 ± 24	29 ± 22	17 ± 13	28 ± 20	48 ± 29	33 ± 29
	**PA**	**AB**	-17 ± 15	-21 ± 18	-24 ± 18	-23 ± 22	-29 ± 25	-25 ± 20	-25 ± 21	-21 ± 18	-22 ± 24	-14 ± 8	-22 ± 20
		**OL**	-20 ± 15	-10 ± 15	-15 ± 16	-18 ± 16	-7 ± 15	-29 ± 20	-2 ± 16	-17 ± 16	-7 ± 14	-7 ± 5	-13 ± 17
		**MAE**	4 ± 2	11 ± 8	10 ± 7	8 ± 8	22 ± 15	6 ± 3	23 ± 13	7 ± 6	15 ± 11	9 ± 6	12 ± 11
	**IS**	**AB**	-113 ± 75	-128 ± 88	-138 ± 98	-123 ± 98	-154 ± 103	-151 ± 114	-146 ± 88	-135 ± 101	-123 ± 90	-138 ± 92	-135 ± 96
		**OL**	-117 ± 73	-122 ± 88	-130 ± 85	-126 ± 85	-156 ± 104	-128 ± 84	-146 ± 86	-134 ± 94	-123 ± 83	-144 ± 51	-133 ± 85
		**MAE**	15 ± 10	14 ± 12	25 ± 27	16 ± 11	20 ± 16	33 ± 36	21 ± 21	16 ± 12	15 ± 15	36 ± 29	21 ± 22
	**R**	**AB**	123 ± 79	144 ± 96	161 ± 114	144 ± 110	181 ± 122	170 ± 125	166 ± 99	150 ± 110	139 ± 101	162 ± 107	154 ± 108
		**OL**	133 ± 71	130 ± 88	135 ± 84	137 ± 86	169 ± 105	145 ± 84	156 ± 85	149 ± 96	131 ± 83	157 ± 50	144 ± 85
		**MAE**	19 ± 13	18 ± 16	37 ± 40	26 ± 16	29 ± 18	41 ± 43	22 ± 25	20 ± 15	19 ± 21	53 ± 32	29 ± 28

For the PFP, the mean resultant HJF of the in vivo measurements is 259%BW ± 33%BW for one-leg stance and 255%BW ± 27%BW for level walking, whereas the mean HJF of the simulations is 280%BW ± 50%BW for one-leg stance and 271%BW ± 33%BW for level walking. This results in the MAPE^PFP^ of 12% ± 14% for one-leg stance and 8% ± 6% for level walking (Table 2). The overestimation for one-leg stance is particularly caused by subject H10R (S2 Table).

**Fig 3:**
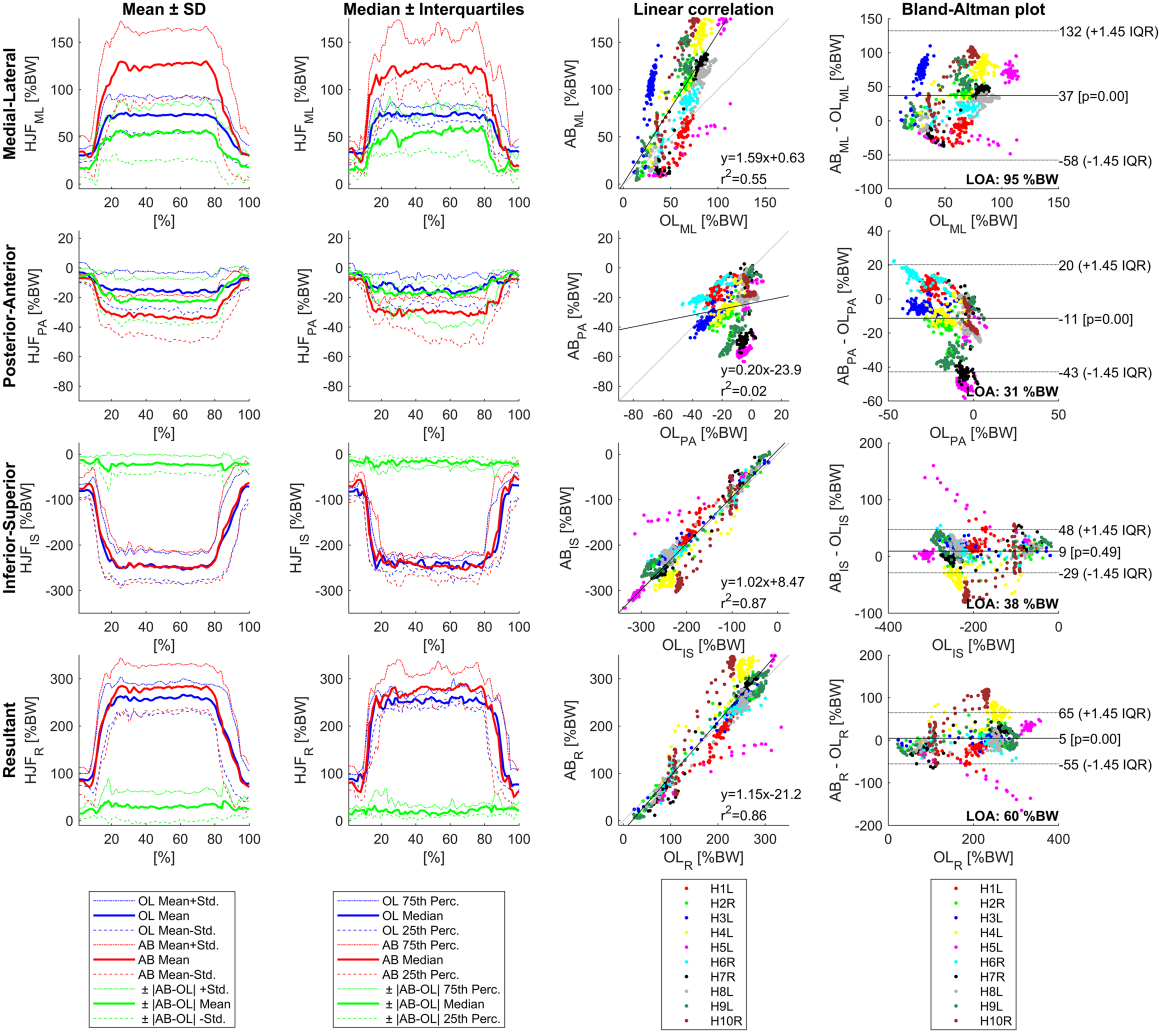
Results for one-leg stance using CT-HJW, 90 N/cm^2^ muscle strength, PN recruitment, and simple muscle model (n = 10).

For the same parameter combination as presented in Table 3, additional statistical analysis was performed for each component and the resultant of the HJF. For one-leg stance, the qualitative comparison of the simulated and the in vivo HJF by the mean and median plots show good agreement over the course of time, whereas the magnitude differs notably in the medial-lateral and posterior-anterior direction (Fig 3). The significant overestimation (p<0.05) of the medial-lateral component is quantified by a slope of 1.59 in the correlation analysis as well as a median difference of 37%BW and LOA of 95%BW in the Bland-Altman plots. Also the posterior-anterior component shows a significant overestimation in the posterior direction of -11%BW (LOA = 31%BW) and posterior-anterior r^2^_PA_ is small (0.02). No significant difference can be observed in the inferior-superior direction, which has the largest r^2^ value of the three components with 0.87 and shows a good correlation with a slope of 1.02 in the correlation analysis. Although the r^2^ value of the resultant HJF is similar to the value of the inferior-superior direction, there is a significant difference (p<0.05) of 5%BW between simulation and in vivo measurements (LOA = 60%BW).

**Fig 4:**
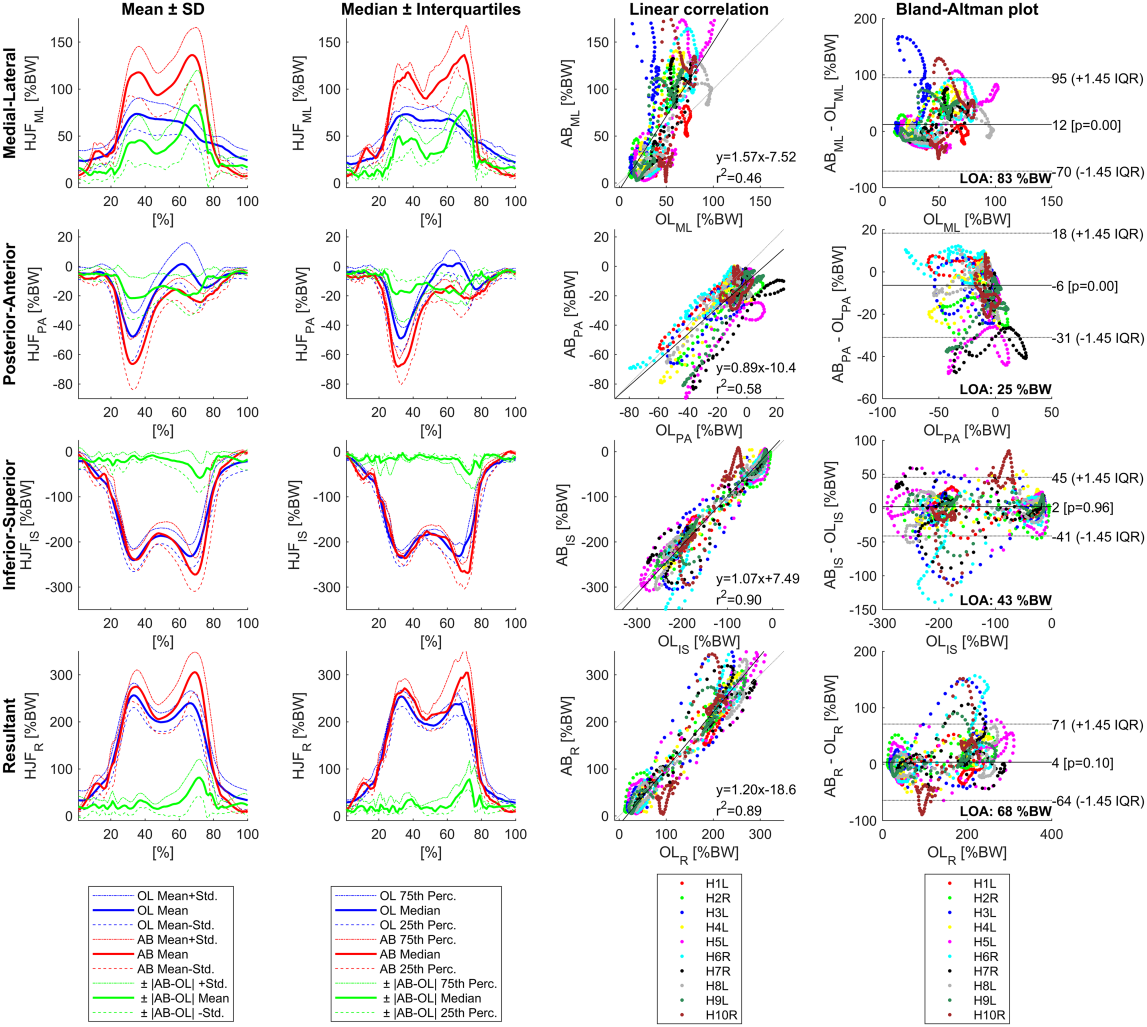
Results for level walking using CT-HJW, 90 N/cm^2^ muscle strength, PN recruitment, and simple muscle model (n = 10).

For level walking, the qualitative comparison of the mean and median curves shows an overestimation of the small peak during ipsilateral heel strike around 10% of the gait cycle in the medial-lateral and inferior-superior direction (Fig 4). For the medial-lateral direction, the distinctive double peak profile of the simulated HJF is not present in the in vivo data and the HJF is significantly overestimated. Values of the correlation analysis and Bland-Altman plots are similar to the values of one-leg stance for the medial-lateral component. For the posterior-anterior component, the HJF is significantly overestimated in the posterior direction. However, r^2^_PA_ (0.58) and slope (0.89) are considerably higher than for one-leg stance and LOA are lower (25%BW). For the inferior-superior component, simulated and in vivo HJF coincide during the contralateral toe off around one third of the gait cycle. However, a small time shift can be observed during contralateral heel strike and the second peak is overestimated. r^2^_IS_ (0.90) and LOA (60%BW) are comparable to the values of one-leg stance for the inferior-superior direction. In contrast to one-leg stance, the difference of 4%BW in the resultant HJF is not significant (LOA = 68%BW).

To evaluate, if the error introduced by the optimization based on the skin markers (Chapter 2.2) correlates with the error between simulated and in vivo HJF, r^2^ was calculated between the difference of C3D-HJW and CT-HJW, and the MAE of the three components and the resultant HJF. There is no significant correlation (α = 0.05) between both parameters, either for one-leg stance or for level walking.

**Fig 5:**
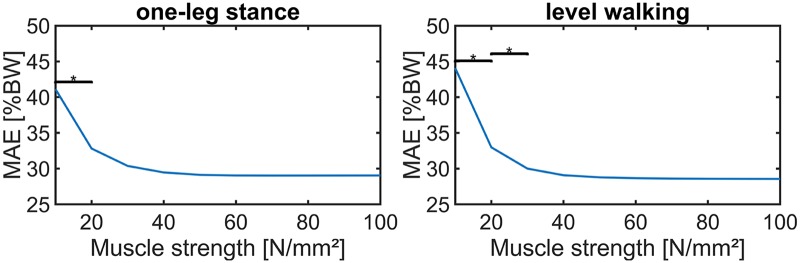
Relationship between muscle strength and the mean absolute error of the resultant HJF (MAE_R_) over the full motion cycle for CT-HJW, PN recruitment, and simple muscle model. Significant differences are marked with an asterisk (*).

Since the two investigated values for muscle strength, 40°N/cm^2^ and 90°N/cm^2^, had a minor impact on the results with CT-HJW, PN recruitment and simple muscle model, the muscle strength was reduced gradually. The MAE_R_ starts to increase significantly if the muscle strength falls below 30°N/cm^2^ (Fig 5).

Finally, simulation times were examined. The mean motion capture times are 8.6 s ± 1.7 s for one-leg stance and 1.8 s ± 0.3 s for level walking. Simulation with PN recruitment took 8 min. to 9 min. for one-leg stance and 2 min. for level walking. This corresponds to a ratio of 1.1 ± 0.1 min. simulation time for 1 second of motion capture time. For the MM recruitment, the ratio of simulation to motion capture time (2.1 ± 0.1 min/s) is twice as high as for the PN recruitment. The impact of muscle strength and muscle model on the simulation time is negligible (S3 Table).

## 4 Discussion

The smallest deviation in magnitude to the in vivo HJF was achieved by using the third power PN recruitment and the simple muscle model. The MM recruitment leads to a general overestimation of the HJF. These results agree with those obtained by Modenese et al., who observed the same effect with increasing power of the polynomial criterion [20]. All simulations with the PN recruitment were successful (Table 2), whereas 11% of the simulations using the MM recruitment failed. Furthermore, simulations with the MM recruitment took twice as long as simulations with the PN recruitment.

Since most of the physiological parameters for the Hill-type muscle model are difficult to obtain for specific muscles, for instance of the lower back [35], the use of a simple muscle model seems to be reasonable to minimize the number of unknown model parameters.

The impact of the muscle strength on the deviations between simulated and in vivo HJF is low. There is no significant difference in MAE_R_ between 30 N/cm^2^ and 100 N/cm^2^ muscle strength in combination with the PN recruitment and the simple muscle model. Above 60 N/cm^2^, none of the muscles reached their maximum force for the two investigated activities (Fig 5). However, this may be different for more demanding activities. For activities of daily living with higher peak forces than one-leg stance and level walking, like stair climbing, it may be advantageous to use a muscle strength of 90 N/cm^2^ in order to avoid that single muscles reach their maximum force. This corresponds with the results of this study, where 90 N/cm^2^ muscle strength achieved the smallest deviations between simulated and in vivo HJF for one-leg stance and level walking. Future research will investigate additional activities of daily living such as stair climbing or sit-to-stand.

The results with CT-HJW are slightly better than the C3D-HJW results based on the skin marker trajectories (Table 2) even if no significant correlation was found between the MAE and the difference of C3D-HJW and CT-HJW. For subjects with a high BMI, the soft tissue between the markers and the bony landmarks in the pelvis region might lead to an overestimation of the HJW during parameter optimization.

The mean deviation in magnitude for the PFP is comparable to the results of previous studies (Table 4). It should be noted that Heller et al. [18] and Zhang et al. [21] did not calculate the mean of the absolute errors, so their deviations might be higher due to cancellations by opposite signs. No results were found in literature relating to the deviation of the orientation during the PFP. In this study, the mean deviations in orientation between the simulated and the in vivo HJF vector were 11° for one-leg stance and 10° for level walking during the PFP. Significant differences of simulated and in vivo HJF over the full motion cycle were found in the medial-lateral and anterior-posterior direction. The highest error occurs in the medial-lateral direction. For level walking, the simulations produced a distinctive double peak profile in the medial-lateral direction that is not present in the in vivo data. This coincides with the results of Zhang et al. [21], who also observed a large difference in the medial-lateral direction.

**Table 4:** Mean deviations between simulation and in vivo peak force results in previous studies for one-leg stance and level walking.

Reference	Software	Subjects	Mean Error Peak Loads [%]	
			One-leg stance	Level walking
Heller et al. [Heller 2001]	Proprietary	4 (HIP98)	NA	12
Stansfield et al. [Stansfield 2003]	Proprietary	2 (HIP98)	10	13
Modenese et al. [Modenese 2011]	OpenSim	4 (HIP98)	NA	10
Zhang et al. [Zhang 2015]	AnyBody	3 (HIP98)	NA	7
This Study	AnyBody	10	12	8

This study comprises a number of limitations. The results need to be interpreted with caution considering the limited number of subjects in the study (n = 10). Only one trial of both activities was available for each subject. Hence, no statement can be made about the variability of the results for repeated motion capture trials of the same subject. In particular, the high overestimation of the resultant HJF during PFP for one-leg stance of subject H10R should be examined with another trial (S2 Table). Subject H10R stands out with the highest BMI of the group (Table 1), but achieves the second lowest resultant HJF during PFP for one-leg stance and the lowest for level walking in the in vivo measurements (S2 Table). For one-leg stance, a high BMI and a low in vivo HJF seem to lead to an overestimation of the simulated HJF by AnyBody for subject H10R. If subject H10R is considered as an outlier and is excluded from the calculation, the MAPE^PFP^ decreases from 12% to 8% for one-leg stance and matches the value for level walking of 8% (Table 4).

Due to the limited number of in vivo data, the validation process was non-blinded because the experimental data was also used in order to find the optimal combination of the four investigated parameters for the clinical application. For a blind validation additional trials of each subject would be necessary [47]. Optimally, the model should be validated against experimental data of additional subjects.

Although the deviations during the PFP are lower for level walking than for one-leg stance, the simulated full gait cycle of level walking shows a time shift and an overestimation of the HJF during contralateral heel strike as well as an underestimation of the HJF during the swing phase (Fig 4). Therefore, it seems that the accuracy of the predicted HJF by AnyBody decreases with increasing motion dynamics. Similar effects were observed in other studies [21,48].

Prospectively, it may be advantageous to use three instead of two force plates for level walking in order to avoid artifacts during the gait cycle caused by the beginning and the end of the force plate contact [48]. Additionally, the effect on the simulated HJF of motion capture trials with arm markers should be investigated. The motion of the arms was not captured for the investigated trials.

In this study, the simulations are based on postoperative motion capture data. Postoperative kinematics might be different from preoperative kinematics [49], and, more importantly, postoperative kinematics might differ from the kinematics of healthy subjects [50,51] due to a protective or relieving posture of the patients. This might lead to a preoperative load-based planning that is not the most optimal for the patient in a long term perspective. An additional drawback of marker-based motion tracking is that the data acquisition during the preoperative planning process is time-consuming, technically difficult, and requires a controlled environment [52,53].

The comparison of electromyography data with simulated muscle activation was not investigated because the focus of the study was to predict the HJF for further use in preoperative planning. In future research, the effect of an electromyography based calibration of muscle recruitment on the prediction of the HJF will be evaluated.

No patient-specific adaption of implant parameters, such as neck length and antetorsion was implemented in order to allow other research groups to use and refer to the exact same model investigated in this study [33]. Moreover, patient-specific parameters like gender, age, fitness level, or the detailed bony morphology were not considered in the model. Only available functionalities of the lower body MoCap-Model were utilized to adapt the model patient-specifically and evaluate the 16 parameter combinations.

## 5 Conclusion & outlook

In conclusion, this study provides important information for the future application of the MSM in preoperative planning of THA. Besides an optimization of the implant alignment during the preoperative planning process, postoperative functional outcome measurements [54] or pre- and postoperative comparisons of the resultant HJF [55] are possible applications of the validated MoCap-Model.

This study has identified the optimal combination of four disputed parameters of MSM to predict the patient-specific HJF. Our research has shown that for one-leg stance and level walking the patient-specifically adapted lower body MoCap-Model of AnyBody predicts the inferior-superior component of the HJF without significant difference compared to the in vivo measurements, whereas significant differences were observed in posterior-anterior and medial-lateral directions. While the impact of the posterior-anterior error on the HJF might be negligible due to the small magnitude of the posterior-anterior component, the deviation in the medial-lateral direction leads to an angular error that would shift the load-based target zone by the same degree. A further study with a focus on the cause of orientation error of the simulated HJF is therefore required. The study will include the patient-specific adaption of the model to consider neck length and antetorsion of the femoral component along with a sensitivity analysis of these parameters. The model will also be validated for other activities of daily living such as stair-climbing or sit-to-stand.

While the automated data pre-processing and the simulation only takes minutes, the acquisition of the motion capture data is more time-consuming. The limitations, which arise from the use of motion capture data, could be addressed in future studies by using models that are based on statistical data [56,57]. These models could include any activities of daily living and be parametrized to take multiple patient-specific parameters into account. The acquisition of the statistical basis for these kinds of models is a comprehensive task. However, the long-term goal will be the substitution of the motion capture-based adaption by means of statistical approaches in order to avoid marker-based motion tracking during the preoperative planning process.

## Supporting information

S1 Figs Simulated vs. in vivo hip joint force. Individual results for one-leg stance and level walking for CT-HJW, 90 N/cm^2^ muscle strength, polynomial muscle recruitment, and simple muscle model.
(PDF)

S1 Table In vivo data. Hip joint force and motion capture file names from the OrthoLoad database used in the study.
(DOCX)

S2 Table Results for the peak force phase. Individual results for one-leg stance and level walking over the peak force phase for CT-HJW, 90 N/cm^2^ muscle strength, polynomial muscle recruitment, and simple muscle model.
(DOCX)

S3 Table Duration of simulations. Mean simulation time and the ratio of simulation to motion capture time for each combination of the four investigated parameters.
(DOCX)

S1 Text List of acronyms. (DOCX)
