# Supplementary material for: Patient-specific musculoskeletal modeling of the hip joint for preoperative planning of total hip arthroplasty: A validation study based on in vivo measurements
Source: PLoS One. 2018 Apr 12;13(4):e0195376. doi: 10.1371/journal.pone.0195376 (PMC5896969; doi:10.1371/journal.pone.0195376)

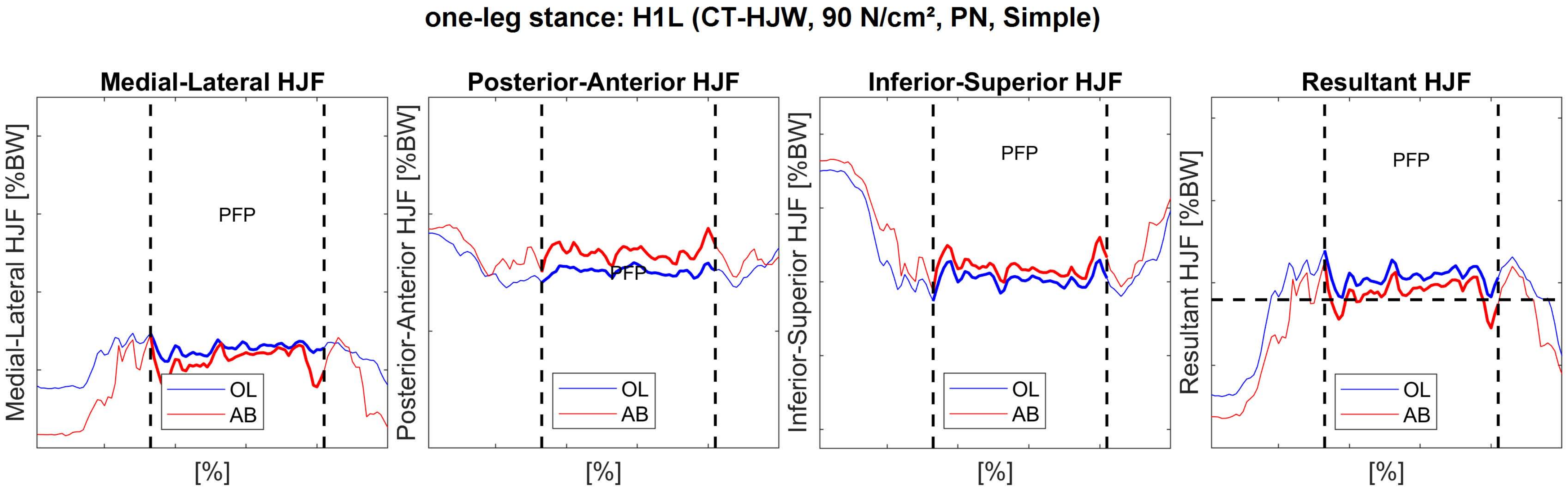

one-leg stance: H1L (CT-HJW, 90 N/cm², PN, Simple)
Medial-Lateral HJF
Medial-Lateral HJF [%BW]
PFP
OL
AB
[%]
Posterior-Anterior HJF
Posterior-Anterior HJF [%BW]
PFP
OL
AB
[%]
Inferior-Superior HJF
Inferior-Superior HJF [%BW]
PFP
OL
AB
[%]
Resultant HJF
Resultant HJF [%BW]
PFP
OL
AB
[%]

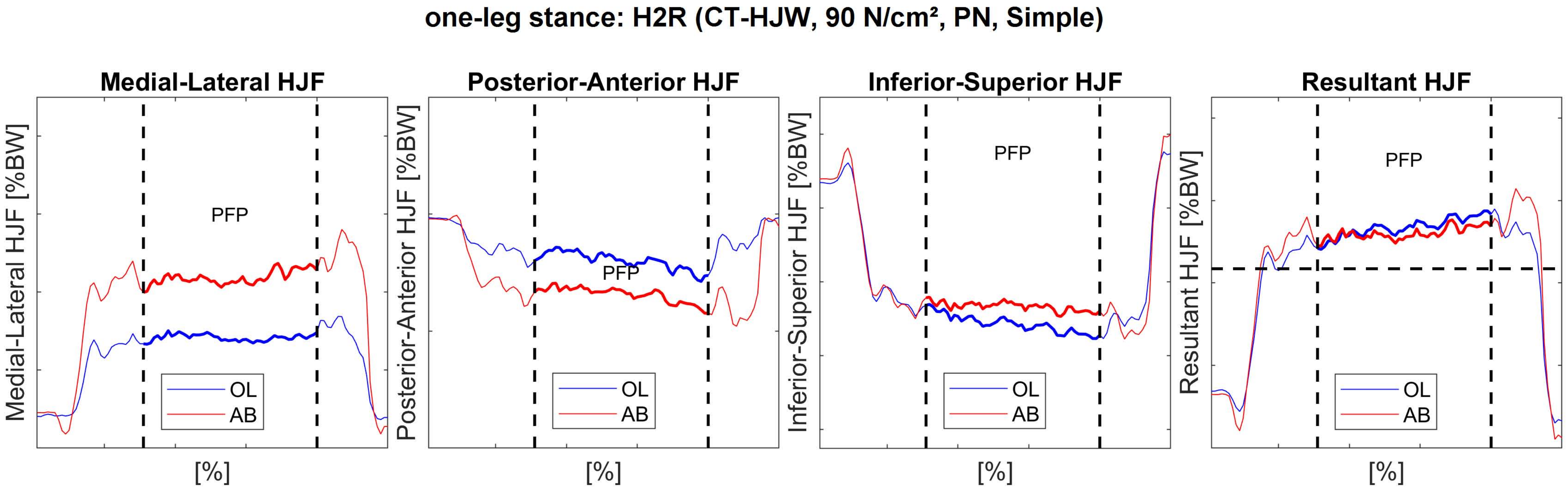
one-leg stance: H2R (CT-HJW, 90 N/cm², PN, Simple)
Medial-Lateral HJF
Medial-Lateral HJF [%BW]
Posterior-Anterior HJF
Posterior-Anterior HJF [%BW]
Inferior-Superior HJF
Inferior-Superior HJF [%BW]
Resultant HJF
Resultant HJF [%BW]
PFP
OL
AB
[%]

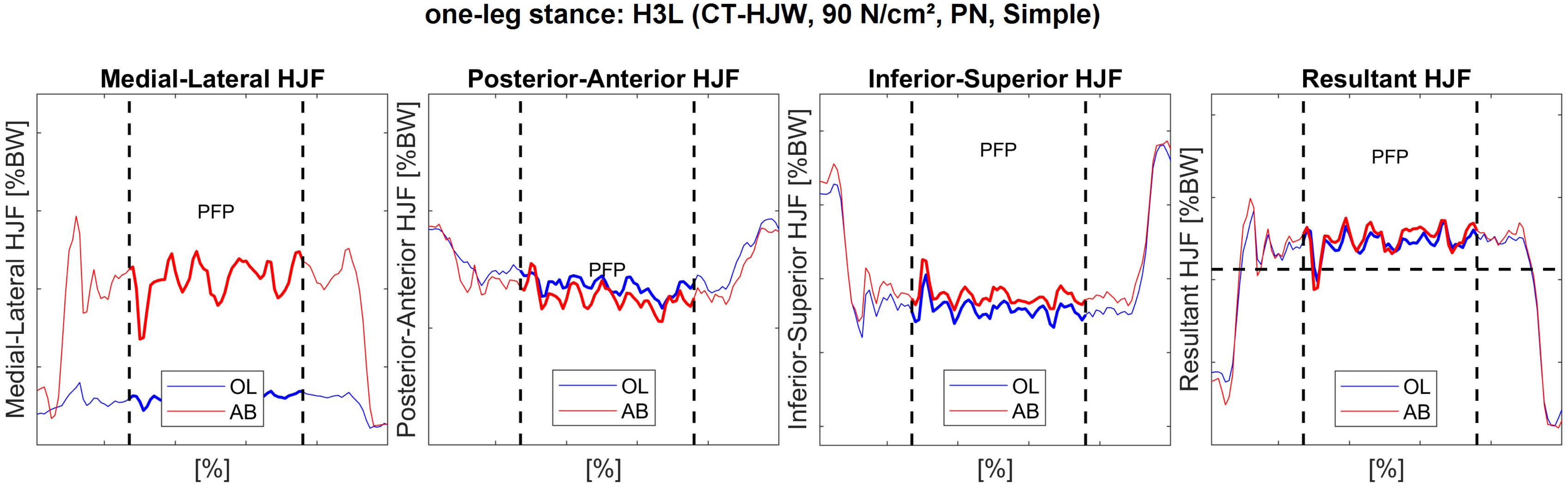
one-leg stance: H3L (CT-HJW, 90 N/cm², PN, Simple)
Medial-Lateral HJF
Medial-Lateral HJF [%BW]
PFP
OL
AB
[%]
Posterior-Anterior HJF
Posterior-Anterior HJF [%BW]
PFP
OL
AB
[%]
Inferior-Superior HJF
Inferior-Superior HJF [%BW]
PFP
OL
AB
[%]
Resultant HJF
Resultant HJF [%BW]
PFP
OL
AB
[%]

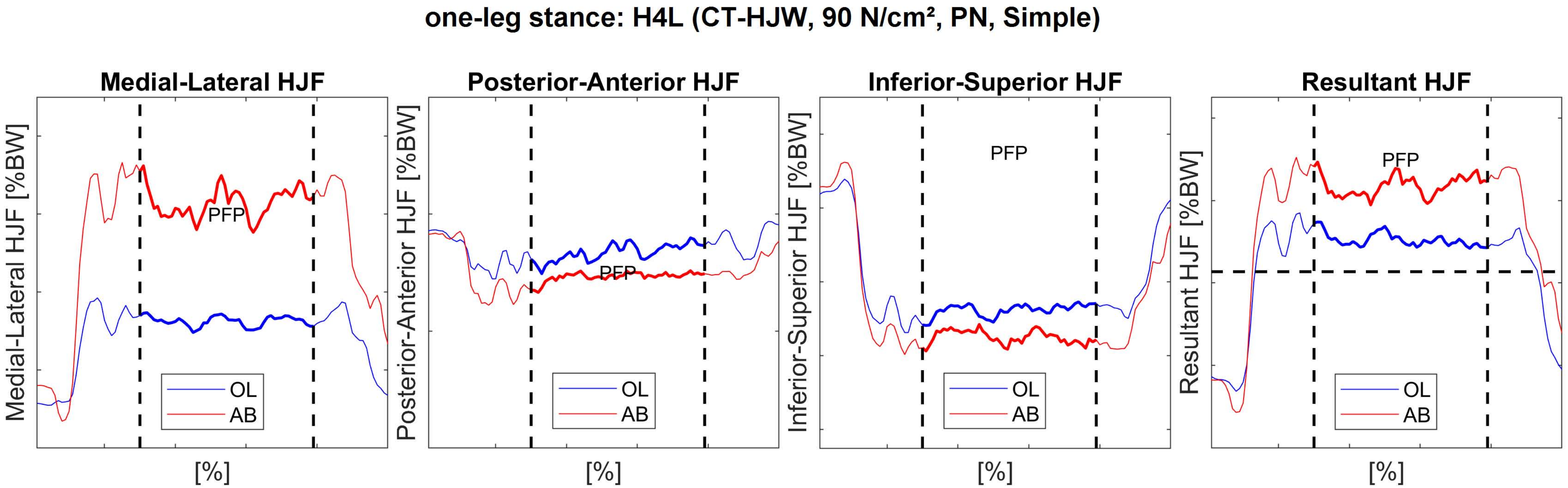

one-leg stance: H4L (CT-HJW, 90 N/cm², PN, Simple)
Medial-Lateral HJF
Medial-Lateral HJF [%BW]
PFP
OL
AB
[%]
Posterior-Anterior HJF
Posterior-Anterior HJF [%BW]
PFP
OL
AB
[%]
Inferior-Superior HJF
Inferior-Superior HJF [%BW]
PFP
OL
AB
[%]
Resultant HJF
Resultant HJF [%BW]
PFP
OL
AB
[%]

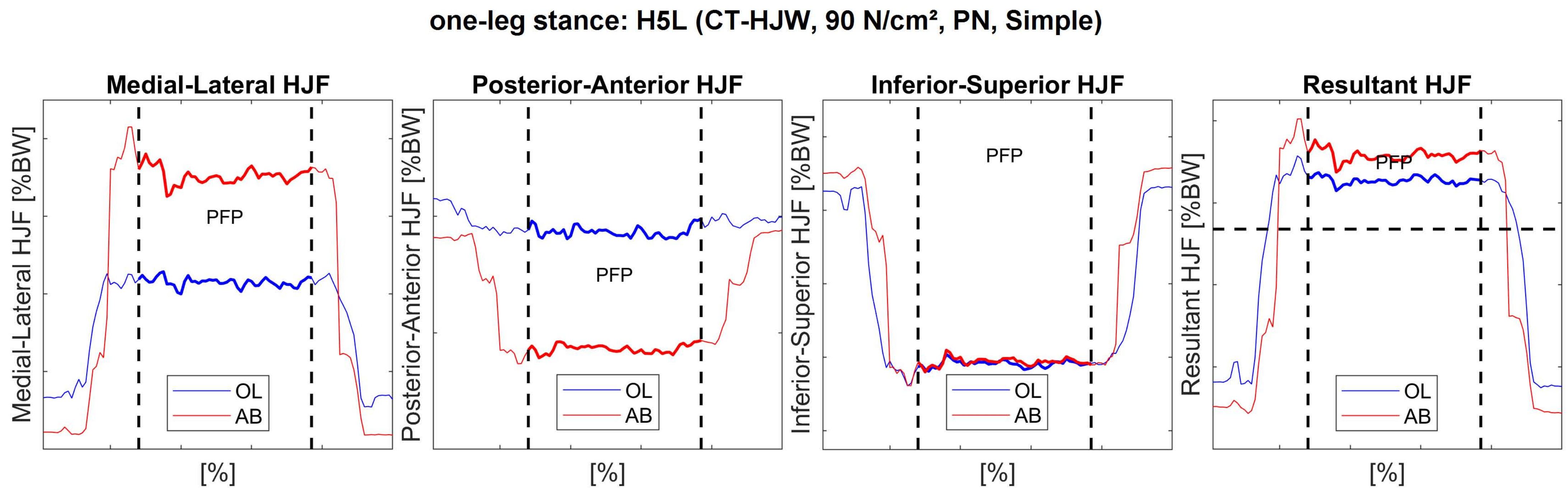

one-leg stance: H5L (CT-HJW, 90 N/cm², PN, Simple)
Medial-Lateral HJF
Medial-Lateral HJF [%BW]
PFP
OL
AB
[%]
Posterior-Anterior HJF
Posterior-Anterior HJF [%BW]
PFP
OL
AB
[%]
Inferior-Superior HJF
Inferior-Superior HJF [%BW]
PFP
OL
AB
[%]
Resultant HJF
Resultant HJF [%BW]
PFP
OL
AB
[%]

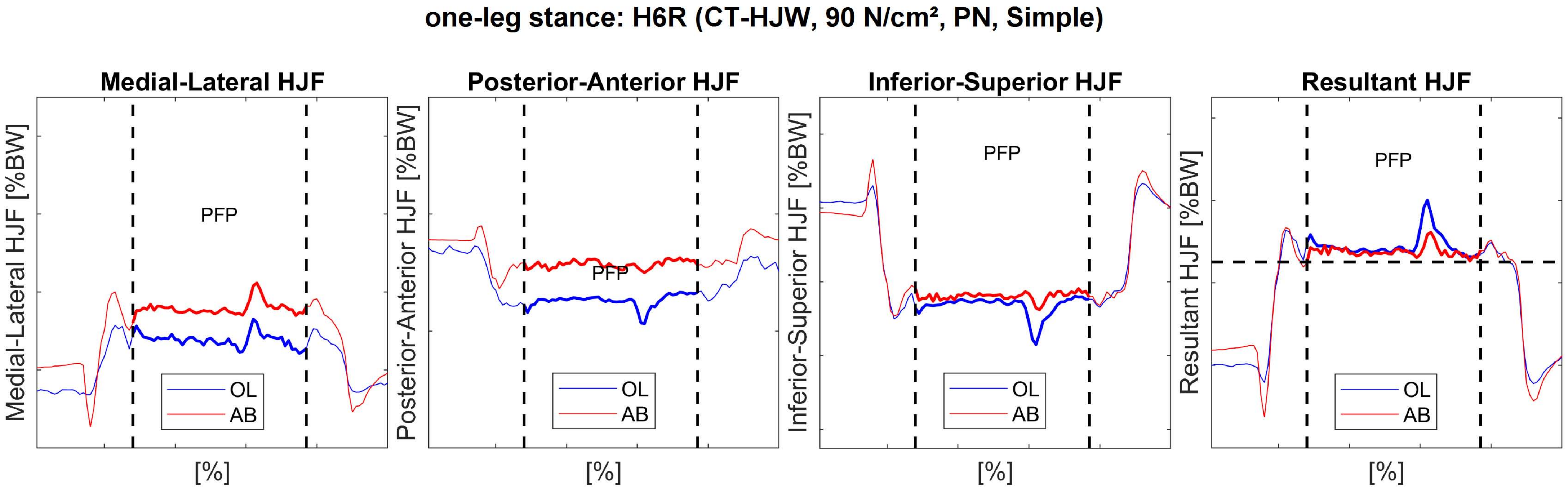
one-leg stance: H6R (CT-HJW, 90 N/cm², PN, Simple)
Medial-Lateral HJF
Medial-Lateral HJF [%BW]
PFP
OL
AB
[%]
Posterior-Anterior HJF
Posterior-Anterior HJF [%BW]
PFP
OL
AB
[%]
Inferior-Superior HJF
Inferior-Superior HJF [%BW]
PFP
OL
AB
[%]
Resultant HJF
Resultant HJF [%BW]
PFP
OL
AB
[%]

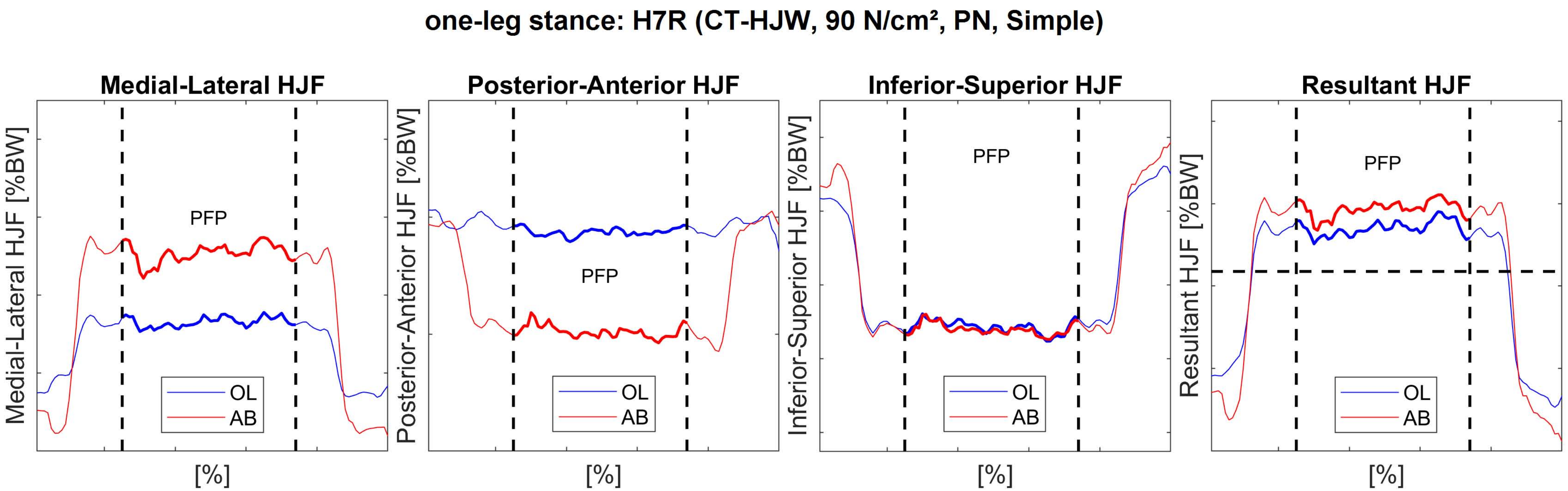
one-leg stance: H7R (CT-HJW, 90 N/cm², PN, Simple)
Medial-Lateral HJF
Medial-Lateral HJF [%BW]
PFP
OL
AB
[%]
Posterior-Anterior HJF
Posterior-Anterior HJF [%BW]
PFP
OL
AB
[%]
Inferior-Superior HJF
Inferior-Superior HJF [%BW]
PFP
OL
AB
[%]
Resultant HJF
Resultant HJF [%BW]
PFP
OL
AB
[%]

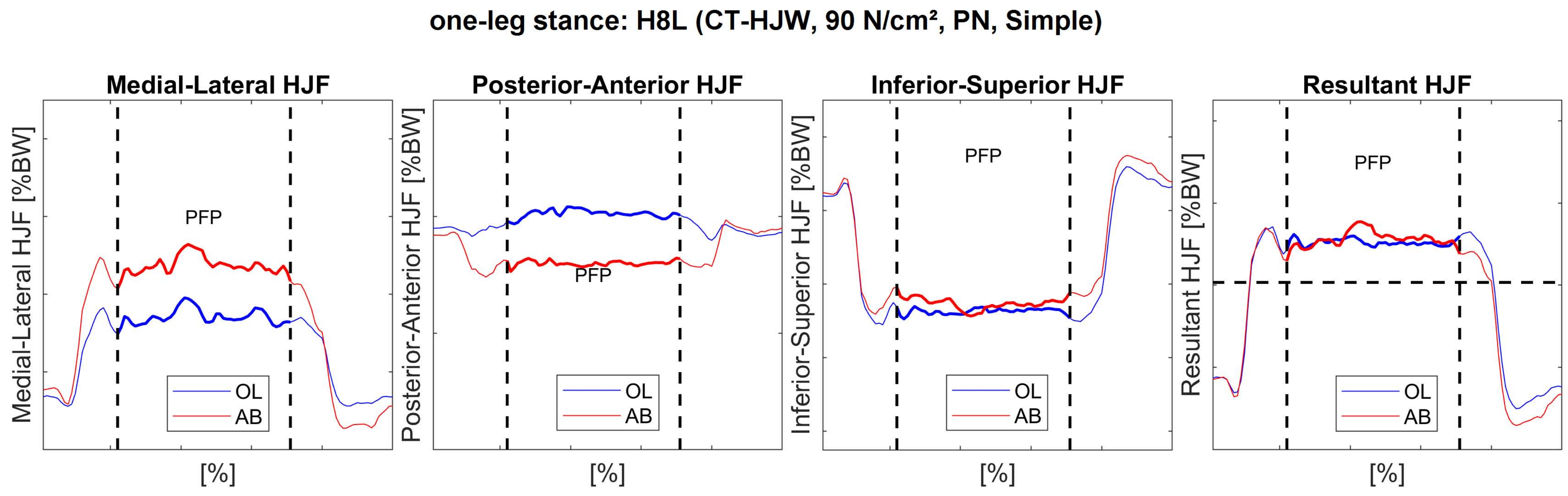
one-leg stance: H8L (CT-HJW, 90 N/cm², PN, Simple)
Medial-Lateral HJF
Medial-Lateral HJF [%BW]
PFP
OL
AB
[%]
Posterior-Anterior HJF
Posterior-Anterior HJF [%BW]
PFP
OL
AB
[%]
Inferior-Superior HJF
Inferior-Superior HJF [%BW]
PFP
OL
AB
[%]
Resultant HJF
Resultant HJF [%BW]
PFP
OL
AB
[%]

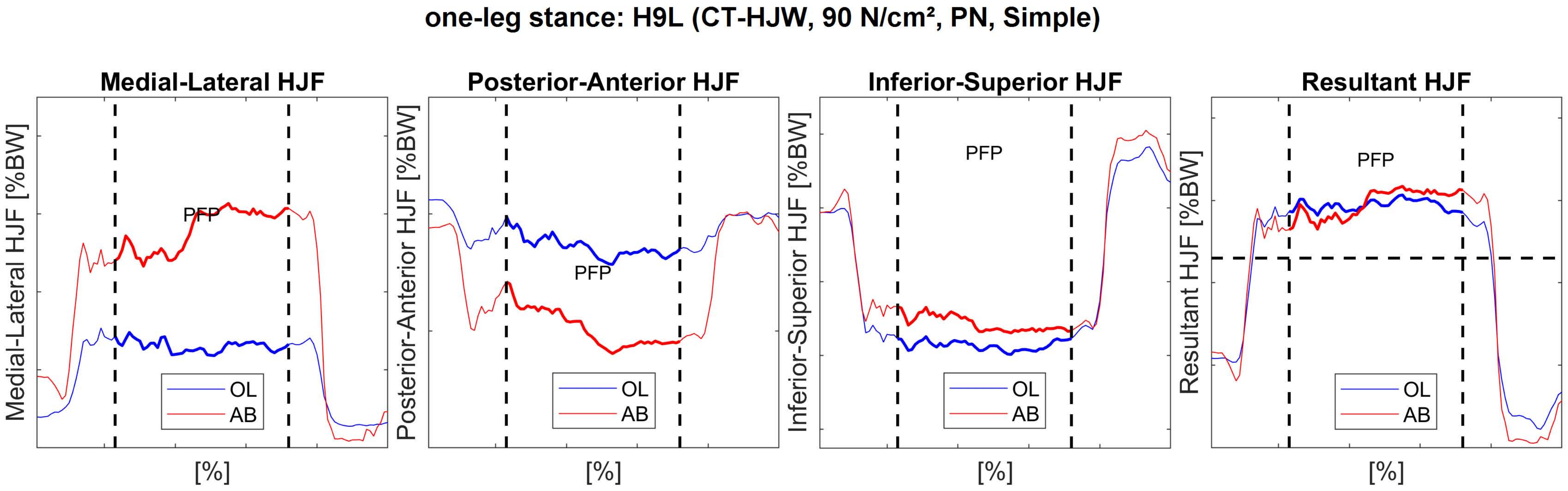

one-leg stance: H9L (CT-HJW, 90 N/cm², PN, Simple)
Medial-Lateral HJF
Medial-Lateral HJF [%BW]
PFP
OL
AB
[%]
Posterior-Anterior HJF
Posterior-Anterior HJF [%BW]
PFP
OL
AB
[%]
Inferior-Superior HJF
Inferior-Superior HJF [%BW]
PFP
OL
AB
[%]
Resultant HJF
Resultant HJF [%BW]
PFP
OL
AB
[%]

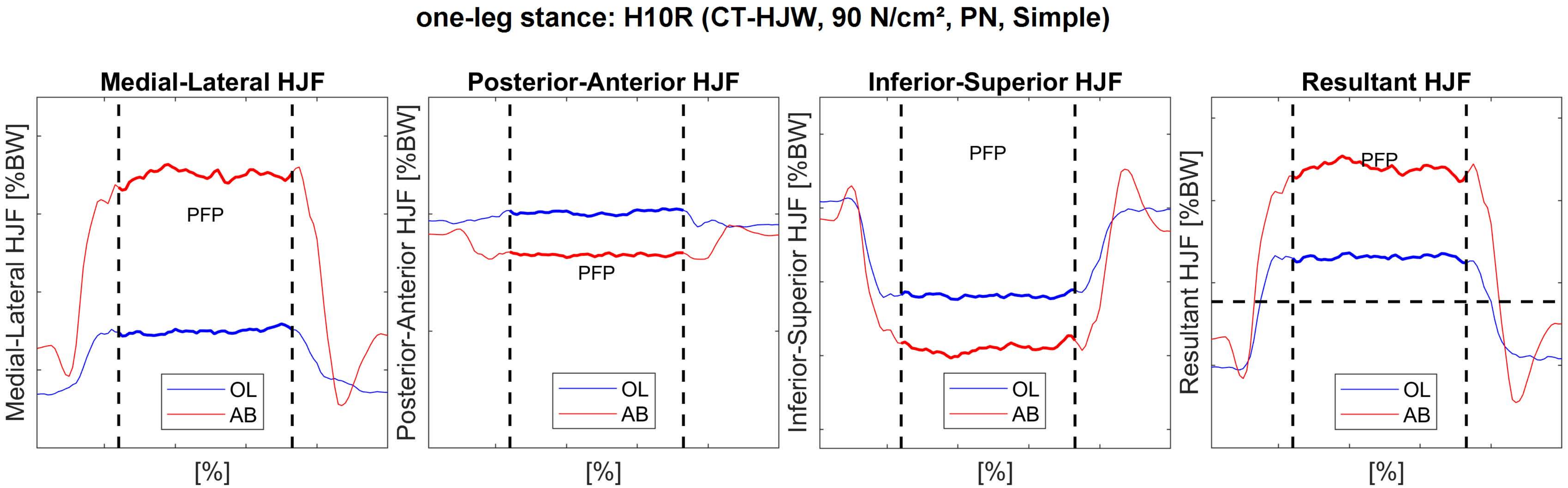
one-leg stance: H10R (CT-HJW, 90 N/cm², PN, Simple)
Medial-Lateral HJF
Medial-Lateral HJF [%BW]
PFP
OL
AB
[%]
Posterior-Anterior HJF
Posterior-Anterior HJF [%BW]
PFP
OL
AB
[%]
Inferior-Superior HJF
Inferior-Superior HJF [%BW]
PFP
OL
AB
[%]
Resultant HJF
Resultant HJF [%BW]
PFP
OL
AB
[%]

level walking: H1L (CT-HJW, 90 N/cm², PN, Simple)

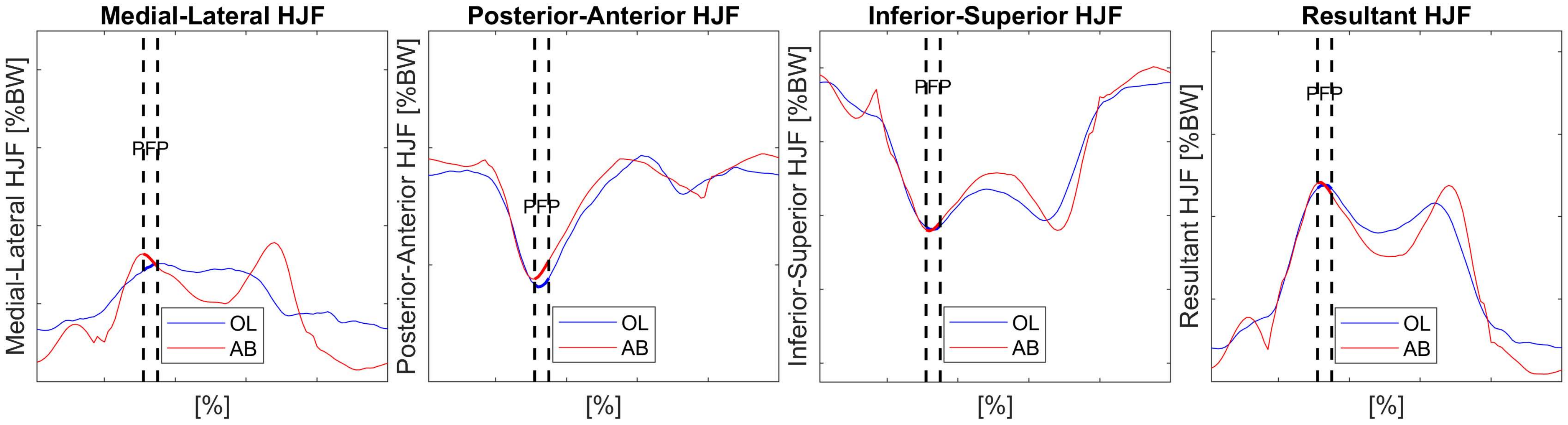

level walking: H2R (CT-HJW, 90 N/cm², PN, Simple)

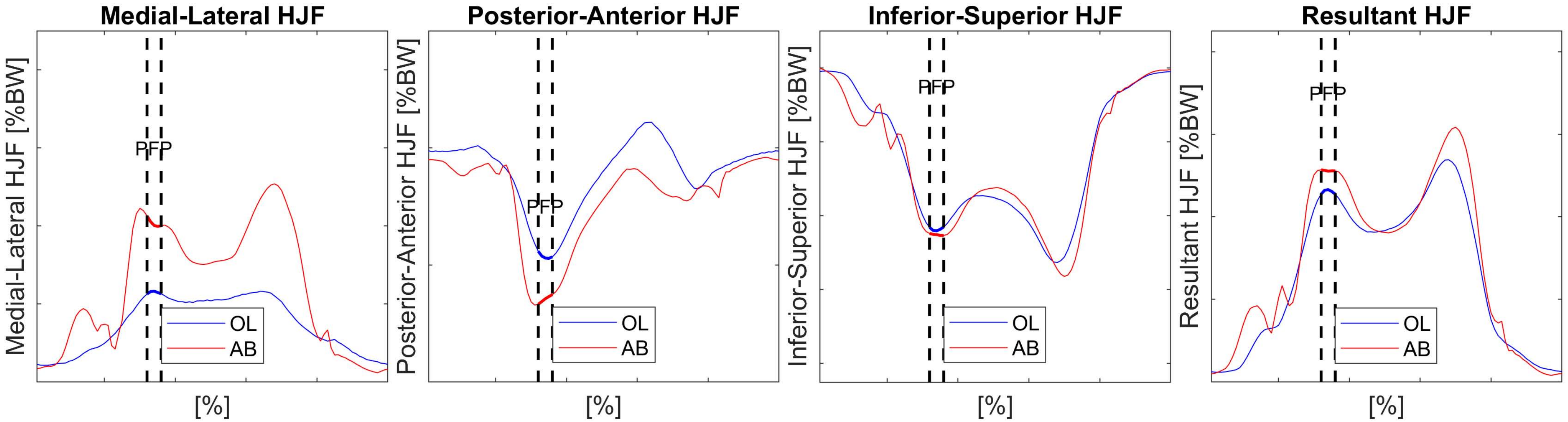

level walking: H3L (CT-HJW, 90 N/cm², PN, Simple)

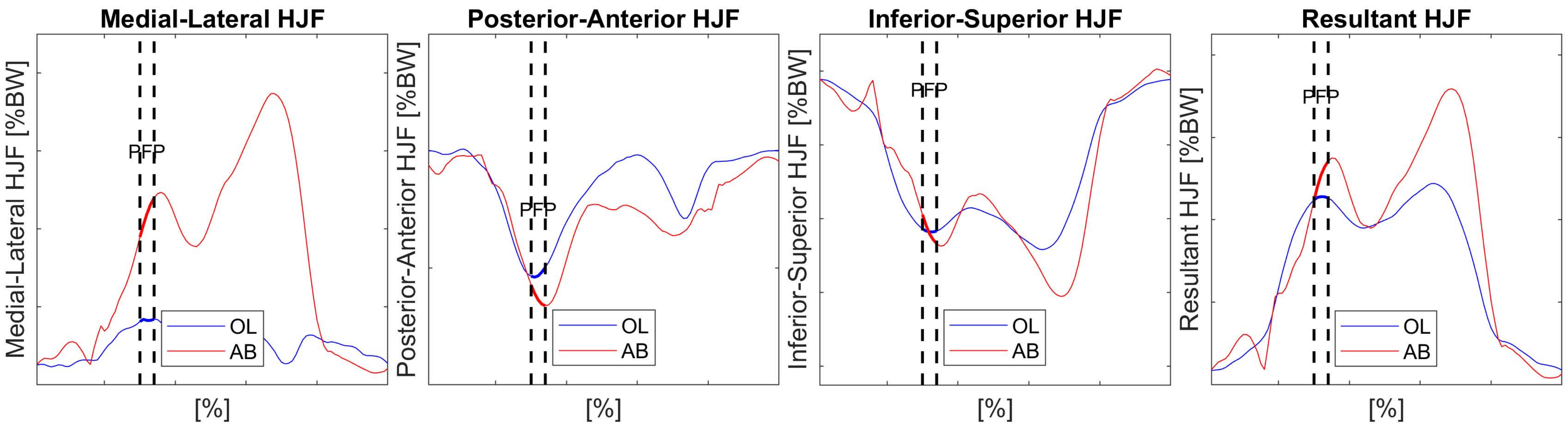

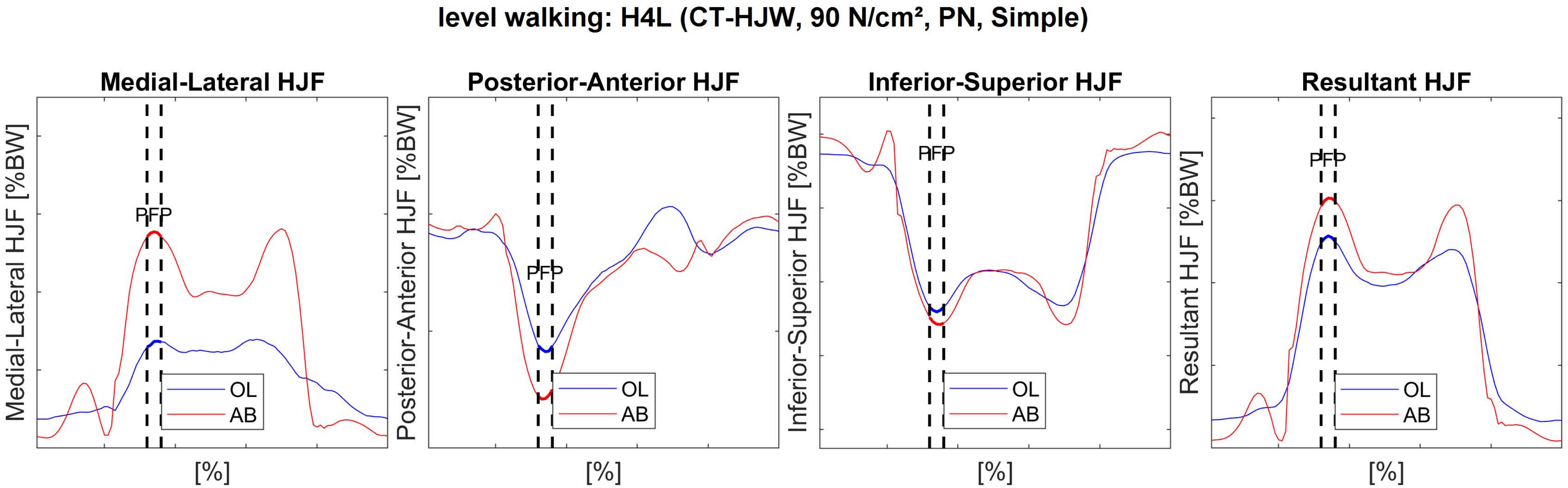

level walking: H4L (CT-HJW, 90 N/cm², PN, Simple)
Medial-Lateral HJF
Medial-Lateral HJF [%BW]
PFP
OL
AB
[%]
Posterior-Anterior HJF
Posterior-Anterior HJF [%BW]
PFP
OL
AB
[%]
Inferior-Superior HJF
Inferior-Superior HJF [%BW]
PFP
OL
AB
[%]
Resultant HJF
Resultant HJF [%BW]
PFP
OL
AB
[%]

level walking: H5L (CT-HJW, 90 N/cm², PN, Simple)

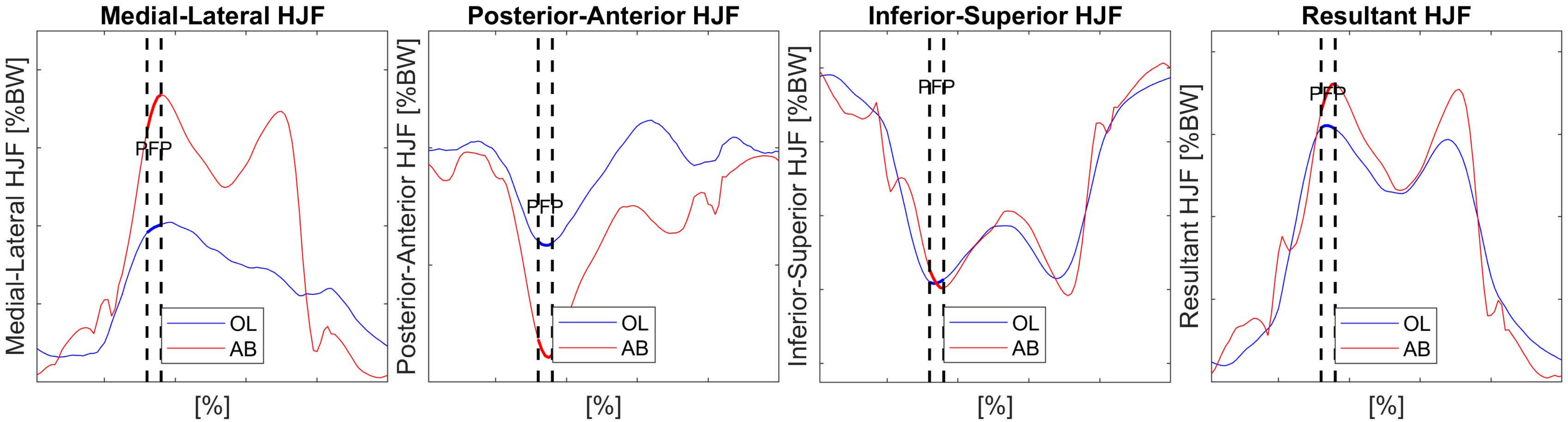

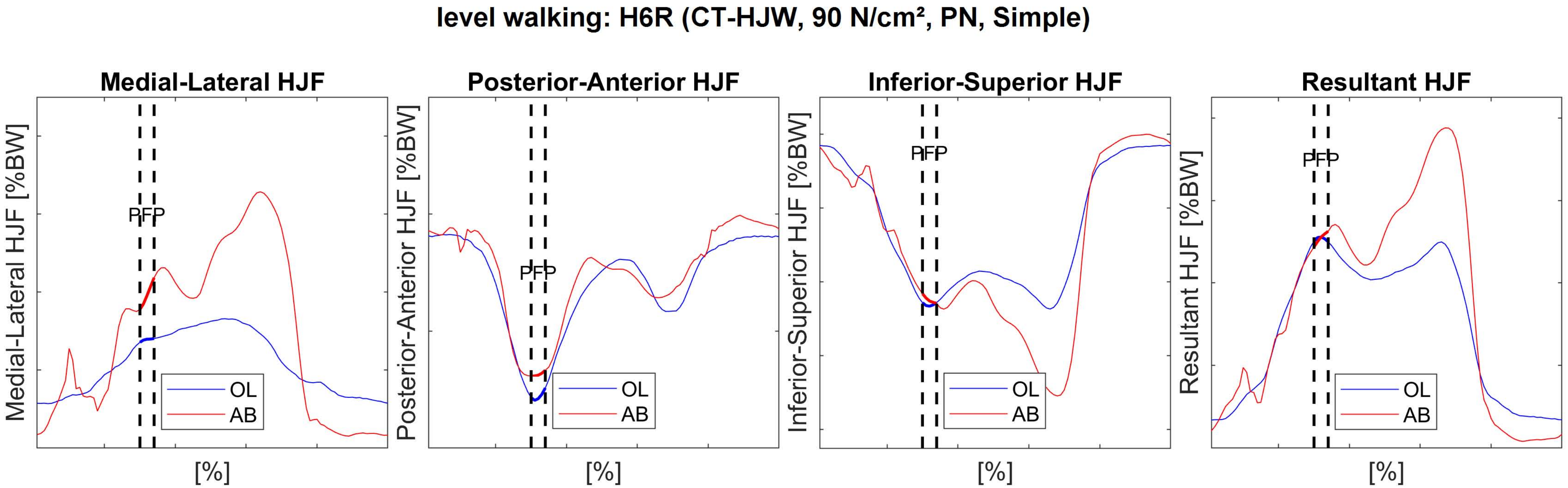
level walking: H6R (CT-HJW, 90 N/cm², PN, Simple)
Medial-Lateral HJF
Medial-Lateral HJF [%BW]
PFP
OL
AB
[%]
Posterior-Anterior HJF
Posterior-Anterior HJF [%BW]
PFP
OL
AB
[%]
Inferior-Superior HJF
Inferior-Superior HJF [%BW]
PFP
OL
AB
[%]
Resultant HJF
Resultant HJF [%BW]
PFP
OL
AB
[%]

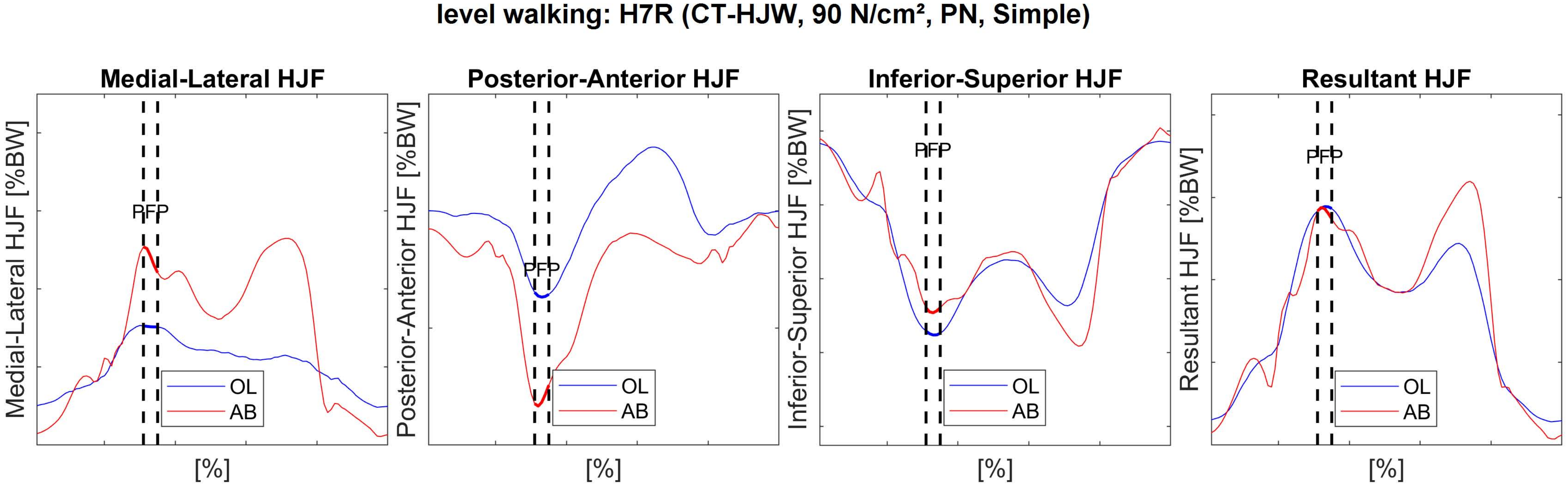
level walking: H7R (CT-HJW, 90 N/cm², PN, Simple)
Medial-Lateral HJF
Medial-Lateral HJF [%BW]
PFP
OL
AB
[%]
Posterior-Anterior HJF
Posterior-Anterior HJF [%BW]
PFP
OL
AB
[%]
Inferior-Superior HJF
Inferior-Superior HJF [%BW]
PFP
OL
AB
[%]
Resultant HJF
Resultant HJF [%BW]
PFP
OL
AB
[%]

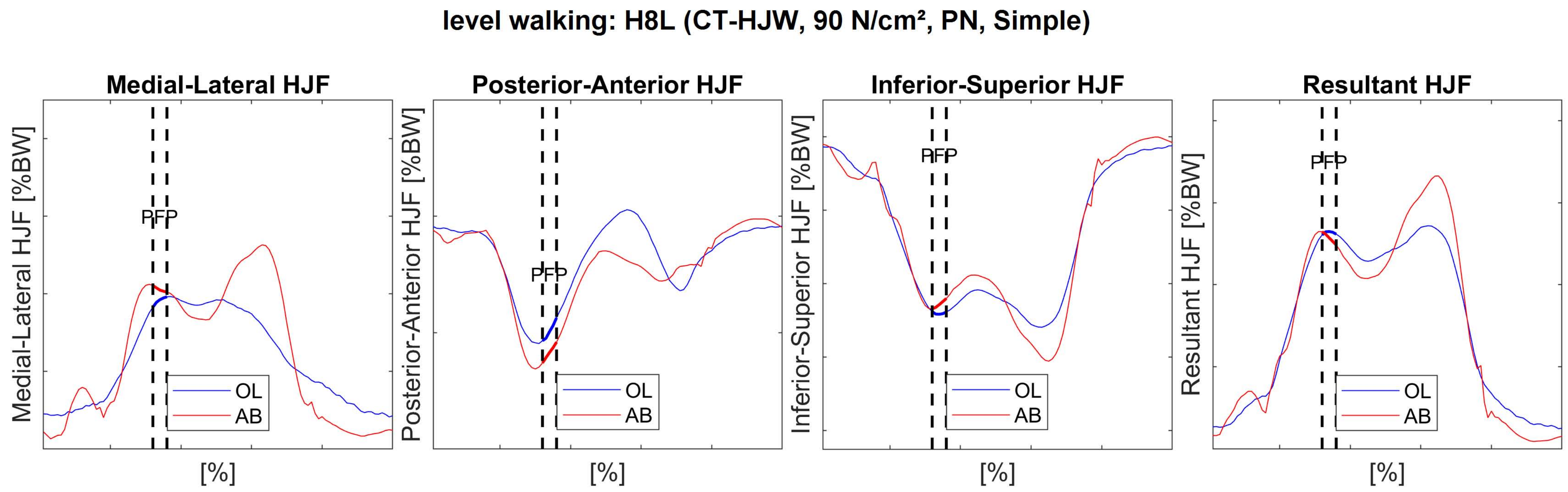
level walking: H8L (CT-HJW, 90 N/cm², PN, Simple)
Medial-Lateral HJF
Medial-Lateral HJF [%BW]
PFP
OL
AB
[%]
Posterior-Anterior HJF
Posterior-Anterior HJF [%BW]
PFP
OL
AB
[%]
Inferior-Superior HJF
Inferior-Superior HJF [%BW]
PFP
OL
AB
[%]
Resultant HJF
Resultant HJF [%BW]
PFP
OL
AB
[%]

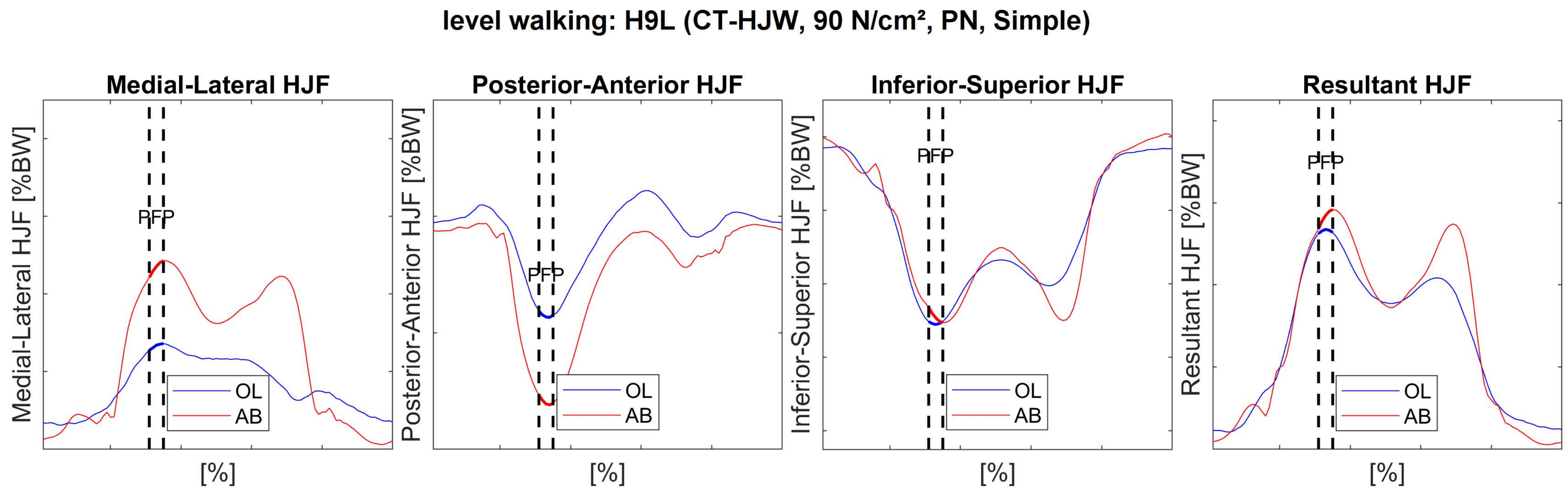
level walking: H9L (CT-HJW, 90 N/cm², PN, Simple)
Medial-Lateral HJF
Medial-Lateral HJF [%BW]
PFP
OL
AB
[%]
Posterior-Anterior HJF
Posterior-Anterior HJF [%BW]
PFP
OL
AB
[%]
Inferior-Superior HJF
Inferior-Superior HJF [%BW]
PFP
OL
AB
[%]
Resultant HJF
Resultant HJF [%BW]
PFP
OL
AB
[%]

level walking: H10R (CT-HJW, 90 N/cm², PN, Simple)

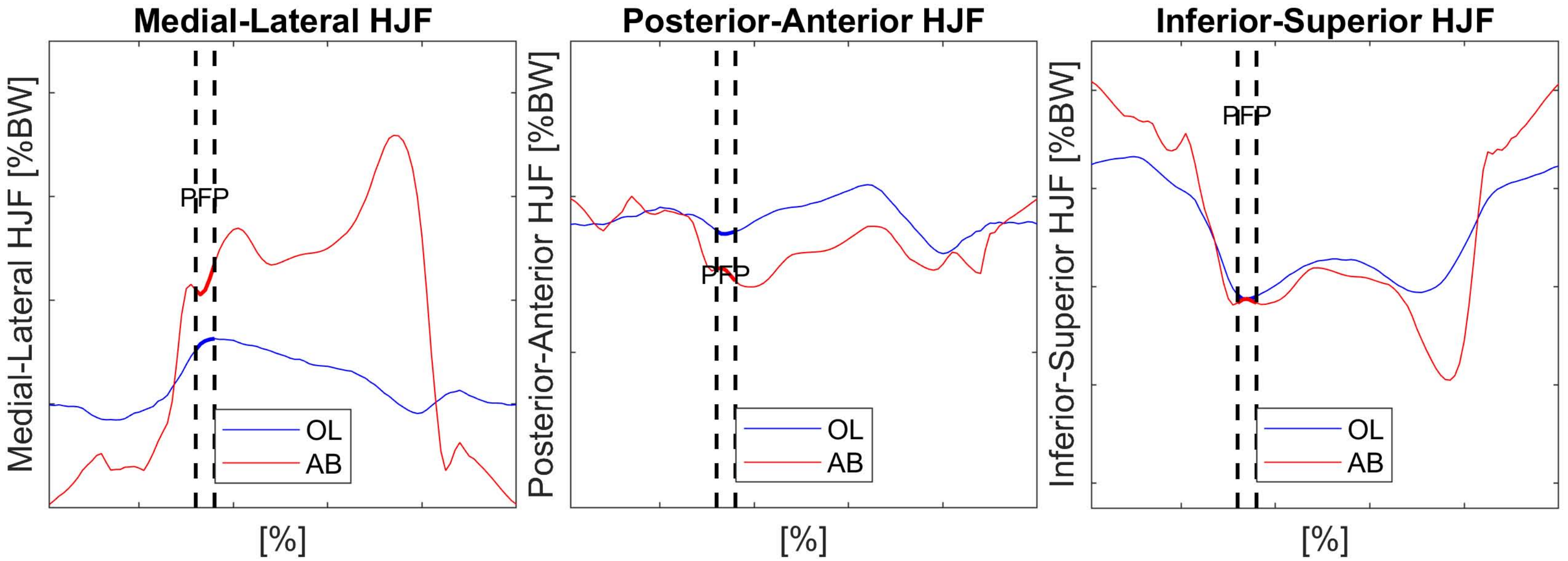

Supplement: S1 Figs — Individual results for one-leg stance and level walking for CT-HJW, 90 N/cm2 muscle strength, polynomial muscle recruitment, and simple muscle model. (PDF) [file pone.0195376.s001.pdf]
