# Supplementary material for: Patient-specific musculoskeletal modeling of the hip joint for preoperative planning of total hip arthroplasty: A validation study based on in vivo measurements
Source: PLoS One. 2018 Apr 12;13(4):e0195376. doi: 10.1371/journal.pone.0195376 (PMC5896969; doi:10.1371/journal.pone.0195376)
Supplement: S1 Table — Hip joint force and motion capture file names from the OrthoLoad database used in the study. (DOCX) [file pone.0195376.s002.docx]

**S1 Table. In vivo data.** Hip joint force and motion capture file names from the OrthoLoad database used in the study.

| **Subject** | **One-leg stance** | **Level walking** |
| --- | --- | --- |
| **H1L** | H1L_060511_1_24 | H1L_060511_1_47 |
| **H2R** | H2R_150811_1_24 | H2R_150811_2_98 |
| **H3L** | H3L_141111_2_75 | H3L_141111_1_28 |
| **H4L** | H4L_270112_1_36 | H4L_270112_2_80 |
| **H5L** | H5L_050412_1_37 | H5L_050412_1_75 |
| **H6R** | H6R_201112_1_36 | H6R_201112_2_107 |
| **H7R** | H7R_191112_1_38 | H7R_191112_1_47 |
| **H8L** | H8L_240413_1_36 | H8L_240413_1_48 |
| **H9L** | H9L_301013_1_29 | H9L_301013_1_62 |
| **H10R** | H10R_300114_1_32 | H10R_300114_1_82 |

Go to: <https://orthoload.com/database/>

- Implant: Hip Joint III
- Activity: Gaitanalysis; Standing; One Legged Stance; Ipsilateral
- Activity: Gaitanalysis; Walking; Level walking
- See “Additional Data” for the motion capture files
