# Supplementary material for: Patient-specific musculoskeletal modeling of the hip joint for preoperative planning of total hip arthroplasty: A validation study based on in vivo measurements
Source: PLoS One. 2018 Apr 12;13(4):e0195376. doi: 10.1371/journal.pone.0195376 (PMC5896969; doi:10.1371/journal.pone.0195376)
Supplement: S2 Table — Individual results for one-leg stance and level walking over the peak force phase for CT-HJW, 90 N/cm2 muscle strength, polynomial muscle recruitment, and simple muscle model. (DOCX) [file pone.0195376.s003.docx]

S2 Table. Results for the peak force phase. Individual results for one-leg stance and level walking over the peak force phase for CT-HJW, 90 N/cm² muscle strength, polynomial muscle recruitment, and simple muscle model. Mean and standard deviation (SD) are presented in %BW for the AnyBody (AB) simulations, the OrthoLoad (OL) in vivo measurements. The mean absolute percentage error (MAPE) of the AnyBody simulations is given in % for each component of the HJF (ML, PA, IS) and the resultant HJF (R).

|  |  |  | **H1L** | **H2R** | **H3L** | **H4L** | **H5L** | **H6R** | **H7R** | **H8L** | **H9L** | **H10R** | **Mean ± SD** |
| --- | --- | --- | --- | --- | --- | --- | --- | --- | --- | --- | --- | --- | --- |
| **One-leg stance** | **ML** | **AB [%BW]** | 56 ± 8 | 109 ± 4 | 107 ± 12 | 158 ± 10 | 176 ± 5 | 90 ± 4 | 127 ± 6 | 118 ± 6 | 140 ± 13 | 175 ± 4 | 126 ± 37 |
|  |  | **OL [%BW]** | 64 ± 4 | 71 ± 2 | 30 ± 2 | 81 ± 3 | 108 ± 3 | 69 ± 4 | 83 ± 3 | 85 ± 5 | 65 ± 4 | 75 ± 2 | 73 ± 19 |
|  |  | **MAPE [%]** | 12 ± 9 | 54 ± 5 | 251 ± 27 | 94 ± 8 | 64 ± 5 | 29 ± 7 | 53 ± 4 | 39 ± 4 | 115 ± 25 | 134 ± 7 | 85 ± 68 |
|  | **PA** | **AB [%BW]** | -16 ± 3 | -34 ± 3 | -37 ± 5 | -27 ± 2 | -57 ± 2 | -21 ± 2 | -49 ± 3 | -20 ± 1 | -49 ± 8 | -17 ± 1 | -33 ± 14 |
|  |  | **OL [%BW]** | -24 ± 2 | -20 ± 4 | -32 ± 4 | -16 ± 4 | -7 ± 2 | -37 ± 3 | -6 ± 2 | 1 ± 2 | -14 ± 5 | 1 ± 1 | -15 ± 13 |
|  |  | **MAPE [%]** | 34 ± 11 | 76 ± 19 | 16 ± 7 | 70 ± 31 | 1003 ± 863 | 42 ± 3 | 754 ± 271 | 3391 ± 9268 | 336 ± 463 | 5043 ± 8178 | 1076 ± 4253 |
|  | **IS** | **AB [%BW]** | -179 ± 14 | -233 ± 6 | -223 ± 13 | -275 ± 9 | -306 ± 5 | -219 ± 6 | -260 ± 8 | -226 ± 8 | -257 ± 11 | -290 ± 6 | -247 ± 37 |
|  |  | **OL [%BW]** | -196 ± 10 | -257 ± 12 | -241 ± 12 | -238 ± 8 | -309 ± 4 | -233 ± 14 | -257 ± 9 | -237 ± 4 | -286 ± 6 | -219 ± 3 | -247 ± 32 |
|  |  | **MAPE [%]** | 8 ± 3 | 9 ± 3 | 7 ± 3 | 15 ± 5 | 1 ± 1 | 5 ± 4 | 2 ± 1 | 5 ± 3 | 10 ± 3 | 33 ± 2 | 10 ± 9 |
|  | **R** | **AB [%BW]** | 189 ± 15 | 260 ± 7 | 251 ± 17 | 318 ± 12 | 358 ± 7 | 238 ± 7 | 294 ± 10 | 256 ± 10 | 296 ± 17 | 339 ± 7 | 280 ± 50 |
|  |  | **OL [%BW]** | 207 ± 11 | 267 ± 12 | 245 ± 12 | 252 ± 8 | 327 ± 5 | 246 ± 14 | 270 ± 9 | 252 ± 4 | 294 ± 6 | 231 ± 3 | 259 ± 33 |
|  |  | **MAPE [%]** | 9 ± 4 | 3 ± 2 | 3 ± 2 | 26 ± 5 | 9 ± 1 | 3 ± 4 | 9 ± 2 | 3 ± 3 | 5 ± 2 | 47 ± 2 | 12 ± 14 |
| **Level walking** | **ML** | **AB [%BW]** | 78 ± 3 | 102 ± 3 | 109 ± 9 | 137 ± 1 | 175 ± 9 | 98 ± 7 | 120 ± 6 | 103 ± 2 | 117 ± 4 | 108 ± 5 | 115 ± 25 |
|  |  | **OL [%BW]** | 74 ± 2 | 57 ± 1 | 41 ± 1 | 67 ± 1 | 99 ± 2 | 69 ± 1 | 76 ± 0 | 95 ± 2 | 66 ± 2 | 80 ± 2 | 72 ± 16 |
|  |  | **MAPE [%]** | 7 ± 5 | 78 ± 6 | 162 ± 20 | 104 ± 4 | 78 ± 5 | 42 ± 9 | 58 ± 8 | 8 ± 4 | 76 ± 2 | 36 ± 5 | 65 ± 45 |
|  | **PA** | **AB [%BW]** | -53 ± 3 | -65 ± 1 | -63 ± 3 | -78 ± 1 | -87 ± 3 | -68 ± 1 | -80 ± 3 | -59 ± 3 | -80 ± 2 | -24 ± 2 | -66 ± 17 |
|  |  | **OL [%BW]** | -58 ± 1 | -46 ± 1 | -53 ± 2 | -58 ± 1 | -41 ± 1 | -78 ± 2 | -36 ± 1 | -49 ± 4 | -42 ± 1 | -12 ± 0 | -47 ± 16 |
|  |  | **MAPE [%]** | 9 ± 3 | 40 ± 6 | 20 ± 9 | 35 ± 2 | 112 ± 4 | 12 ± 1 | 122 ± 10 | 19 ± 3 | 88 ± 1 | 112 ± 18 | 57 ± 44 |
|  | **IS** | **AB [%BW]** | -217 ± 4 | -226 ± 1 | -217 ± 14 | -255 ± 3 | -289 ± 9 | -224 ± 5 | -243 ± 3 | -228 ± 5 | -244 ± 8 | -215 ± 2 | -236 ± 23 |
|  |  | **OL [%BW]** | -217 ± 2 | -218 ± 2 | -217 ± 1 | -237 ± 3 | -290 ± 2 | -230 ± 2 | -274 ± 2 | -240 ± 1 | -253 ± 2 | -211 ± 2 | -239 ± 25 |
|  |  | **MAPE [%]** | 1 ± 1 | 4 ± 1 | 5 ± 3 | 7 ± 1 | 3 ± 2 | 3 ± 2 | 12 ± 1 | 5 ± 2 | 4 ± 3 | 2 ± 1 | 5 ± 3 |
|  | **R** | **AB [%BW]** | 236 ± 6 | 256 ± 1 | 251 ± 17 | 299 ± 3 | 349 ± 13 | 254 ± 7 | 282 ± 5 | 257 ± 6 | 282 ± 9 | 242 ± 4 | 271 ± 33 |
|  |  | **OL [%BW]** | 236 ± 2 | 230 ± 2 | 227 ± 1 | 253 ± 3 | 309 ± 2 | 253 ± 2 | 287 ± 2 | 263 ± 2 | 265 ± 2 | 226 ± 2 | 255 ± 26 |
|  |  | **MAPE [%]** | 2 ± 1 | 11 ± 1 | 11 ± 7 | 18 ± 1 | 13 ± 4 | 2 ± 2 | 2 ± 2 | 3 ± 2 | 6 ± 3 | 7 ± 2 | 8 ± 6 |
