# Supplementary material for: Patient-specific musculoskeletal modeling of the hip joint for preoperative planning of total hip arthroplasty: A validation study based on in vivo measurements
Source: PLoS One. 2018 Apr 12;13(4):e0195376. doi: 10.1371/journal.pone.0195376 (PMC5896969; doi:10.1371/journal.pone.0195376)
Supplement: S3 Table — Mean simulation time and the ratio of simulation to motion capture time for each combination of the four investigated parameters. (DOCX) [file pone.0195376.s004.docx]

**S3 Table. Duration of simulations.** Mean simulation time and the ratio of simulation to motion capture time for each combination of the four investigated parameters.

| **Hip joint width** | **Muscle strength [N/cm²]** | **Muscle recruitment** | **Muscle model** | **One-leg stance** | | **Level walking** | |
| --- | --- | --- | --- | --- | --- | --- | --- |
|  |  |  |  | **Simulation time [min]** | **Simulation to motion capture time ratio [min/s]** | **Simulation time [min]** | **Simulation to motion capture time ratio [min/s]** |
| **C3D** | **40** | **PN** | **Simple** | 8 ± 2 | 0.9 | 2 ± 0 | 1.1 |
|  |  |  | **Hill** | 8 ± 1 | 1 | 2 ± 0 | 1.2 |
|  |  | **MM** | **Simple** | 17 ± 3 | 2 | 4 ± 1 | 2.2 |
|  |  |  | **Hill** | 17 ± 3 | 1.9 | 4 ± 1 | 2.2 |
|  | **90** | **PN** | **Simple** | 8 ± 2 | 1 | 2 ± 0 | 1.1 |
|  |  |  | **Hill** | 9 ± 2 | 1 | 2 ± 0 | 1.2 |
|  |  | **MM** | **Simple** | 16 ± 2 | 1.9 | 4 ± 0 | 2.1 |
|  |  |  | **Hill** | 17 ± 3 | 1.9 | 4 ± 1 | 2.3 |
| **CT** | **40** | **PN** | **Simple** | 8 ± 1 | 0.9 | 2 ± 0 | 1.1 |
|  |  |  | **Hill** | 8 ± 1 | 1 | 2 ± 0 | 1.2 |
|  |  | **MM** | **Simple** | 17 ± 3 | 2 | 4 ± 1 | 2.1 |
|  |  |  | **Hill** | 17 ± 3 | 1.9 | 4 ± 1 | 2.2 |
|  | **90** | **PN** | **Simple** | 8 ± 1 | 1 | 2 ± 0 | 1.1 |
|  |  |  | **Hill** | 9 ± 1 | 1 | 2 ± 0 | 1.2 |
|  |  | **MM** | **Simple** | 19 ± 4 | 2.2 | 4 ± 1 | 2.2 |
|  |  |  | **Hill** | 17 ± 3 | 2 | 4 ± 1 | 2.3 |
