# Supplementary material for: Patient-specific musculoskeletal modeling of the hip joint for preoperative planning of total hip arthroplasty: A validation study based on in vivo measurements
Source: PLoS One. 2018 Apr 12;13(4):e0195376. doi: 10.1371/journal.pone.0195376 (PMC5896969; doi:10.1371/journal.pone.0195376)
Supplement: S1 Text — (DOCX) [file pone.0195376.s005.docx]

# List of acronyms

| AA | Adduction-abduction |
| --- | --- |
| AB | AnyBody (simulation results) |
| %BW | Percentage of body weight |
| C3D | Coordinate three dimensional: the file format of the motion tracking and force plate data |
| C3D-HJW | C3D-based hip joint with: the hip joint width optimized based on the motion tracking data |
| CT | Computed tomography |
| CT-HJW | CT-based hip joint width: the hip joint width measured in the CT images |
| FE | Flexion-extension |
| HJF | Hip joint force |
| HJW | Hip joint width |
| IE | Internal-external rotation |
| IQR | Interquartile range |
| IS | Inferior-Superior (HJF) |
| LOA | Limits of agreement |
| MAD | Mean angular deviation |
| MAE | Mean absolute error |
| MAPE | Mean absolute percentage error |
| ML | Medial-lateral (HJF) |
| MM | Strict min/max muscle recruitment criterion |
| MSM | Musculoskeletal models |
| OL | OrthoLoad (in vivo measurements) |
| PFP | Peak force phase |
| PN | Polynomial muscle recruitment criterion |
| PS | Posterior-anterior (HJF) |
| R | Resultant (HJF) |
| r² | Squared Pearson correlation coefficient |
| RMSE | Root mean square error |
| SD | Standard deviation |
| THA | Total hip arthroplasty |
